# Solvent-Responsive Luminescence of an 8-Hydroxyquinoline-Modified 1*H*-Imidazo[4,5-*f*][1,10]phenanthroline Ligand and Its Cu(I) Complexes: Excited-State Mechanisms and Structural Effects

**DOI:** 10.3390/molecules30193973

**Published:** 2025-10-03

**Authors:** Zhenqin Zhao, Siyuan Liu, Shu Cui, Yichi Zhang, Ziqi Jiang, Xiuling Li

**Affiliations:** School of Chemistry and Materials Science, Jiangsu Normal University, Xuzhou 221116, China3020210913@jsnu.edu.cn (Y.Z.);

**Keywords:** copper(I) complexes, 1*H*-imidazo[4,5-*f*][1,10]phenanthroline, 8-Hydroxyquinoline, solvent-responsive luminescence, excited-state mechanism

## Abstract

Understanding how solvents influence the luminescence behavior of Cu(I) complexes is crucial for designing advanced optical sensors. This study reports the synthesis, structures and photophysical investigation of an 8-hydroxyquinoline-functionalized 1*H*-imidazo[4,5-*f*][1,10]phenanthroline ligand, ipqH_2_, and its four Cu(I) complexes with diphosphine co-ligands. Photoluminescence studies demonstrated distinct solvent-dependent excited-state mechanisms. In DMSO/alcohol mixtures, free ipqH_2_ exhibited excited-state proton transfer (ESPT) and enol-keto tautomerization, producing dual emission at about 447 and 560 nm, while the complexes resisted ESPT due to hydrogen bond blocking by PF_6_^−^ anions and Cu(I) coordination. In DMSO/H_2_O, aggregation-caused quenching (ACQ) and high-energy O–H vibrational quenching dominated, but complexes **1** and **2** showed a significant red-shifted emission (569–574 nm) with high water content due to solvent-stabilized intra-ligand charge transfer and metal-to-ligand charge transfer ((IL+ML)CT) states. In DMSO/DMF, hydrogen bond competition and solvation-shell reorganization led to distinct responses: complexes **1** and **3**, with flexible bis[(2-diphenylphosphino)phenyl]ether (POP) ligands, displayed peak splitting and (IL + ML)CT redshift emission (501 ⟶ 530 nm), whereas complexes **2** and **4**, with rigid 9,9-dimethyl-4,5-bis(diphenylphosphino)-9*H*-xanthene (xantphos), showed weaker responses. The flexibility of the diphosphine ligand dictated DMF sensitivity, while the coordination, the hydrogen bonds between PF_6_^−^ anions and ipqH_2_, and water solubility governed the alcohol/water responses. This work elucidates the multifaceted solvent-responsive mechanisms in Cu(I) complexes, facilitating the design of solvent-discriminative luminescent sensors.

## 1. Introduction

The 21st century has witnessed remarkable advancements in optical functional materials, expanding their applications from basic lighting to intelligent sensing, bioimaging, and phototherapy [1,2,3]. Among these, luminescent transition metal complexes have attracted significant attention due to their large Stokes shifts and long-lived phosphorescence. Although noble metal Au(I) [4,5], Pt(II) [6,7], and Ir(III) complexes exhibit enhanced phosphorescence through strong spin–orbit coupling, their high cost limits their widespread application [2,8,9]. In contrast, cuprous complexes have emerged as promising alternatives, offering cost-effectiveness, environmental friendliness, and tunable photophysical properties [10], particularly those based on imidazo[1,10]phenanthroline (Imphens) and diphosphine ligands [11,12,13,14,15,16,17].

On the other hand, solvent-responsive luminescent materials dynamically modulated their emissions in response to solvent microenvironments, governed by multifaceted mechanisms such as solvent polarity [18,19], excited-state proton transfer (ESPT) [19,20,21,22,23], charge transfer (CT) [23], solvent coordination [24], etc. In 2023, Pan’s group presented a novel design for synthesizing pure organic photoluminescent molecules with dual excited-state intramolecular proton transfer (ESIPT) sites, enabling full-color emission and white light in various solvents [22]. In addition, many carbon dots (CDs) also exhibited significant solvent effects [23]. In 2021, CDs prepared from ninhydrin and *o*-phenylenediamine displayed solvent-dependent green-to-red fluorescence changes caused by hydrogen bonding between surface hydroxyl/amino/carbonyl groups and solvents, and these CDs were successfully applied in white-light LEDs and solvent water-content detection [25].

From the above studies, it is evident that the current research for solvent-responsive luminescence is primarily focused on organic molecules and nanoparticles, with limited exploration of luminescent complexes. Solvent-responsive luminescence in Cu(I) complexes remains underexplored. Developing and investigating such complexes can not only elucidate their excited-state regulation principles but also provide theoretical support for designing bioprobes with large Stokes shift and chemical sensors with high sensitivity, which holds significant scientific value for advancing intelligent sensing materials. Herein, 2*-*(1*H*-imidazo[4,5-*f*][1,10]phenanthrolin-2-yl)quinolin-8-ol (ipqH_2_) and its four Cu(I) complexes were synthesized, and their solvent-responsive luminescent mechanisms were elucidated.

## 2. Results and Discussion

### 2.1. Syntheses

**Scheme 1:**
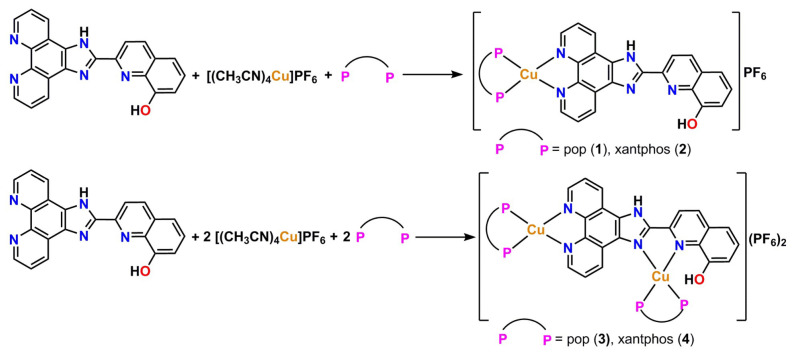
Synthetic routes of the Cu(I) complexes.

The synthesis process of the ligand ipqH_2_ has been slightly modified according to the literature [26]. No triethyl orthoformate was added during the synthesis process, and the product precipitate was obtained by adding *n*-hexane instead of water to concentrated ethanol solution in the post-treatment phase. The synthesis strategy of the Cu(I) complexes follows the methods in classical studies [11,12,13,14,15,16]. At room temperature, ipqH_2_ and [Cu(CH_3_CN)_4_]PF_6_ was reacted with diphosphine ligand bis[(2-diphenylphosphino)phenyl]ether (POP) or 9,9-dimethyl-4,5-bis(diphenylphosphino)-9*H*-xanthene (xantphos) in CH_2_Cl_2_ solutions at molar ratios of 1:1:1 and 1:2:2, respectively (see Scheme 1), and the crystals were then cultured in a mixed solvent of CH_2_Cl_2_/*n*-hexane.

### 2.2. X-Ray Structure Analysis

**Figure 1:**
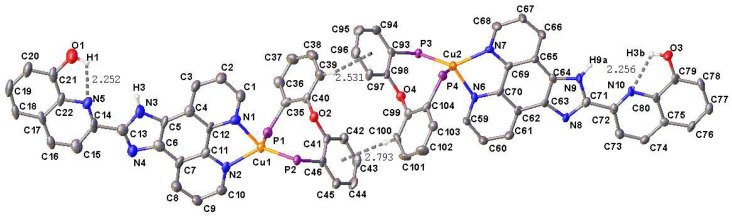
ORTEP drawing of the cation dimer of complex **1** in the asymmetric unit with thermal ellipsoids at 50% probability. For clarity, most of the hydrogen atoms and the benzene rings in the PPh_2_ units of the POP ligand have been omitted.

**Figure 2:**
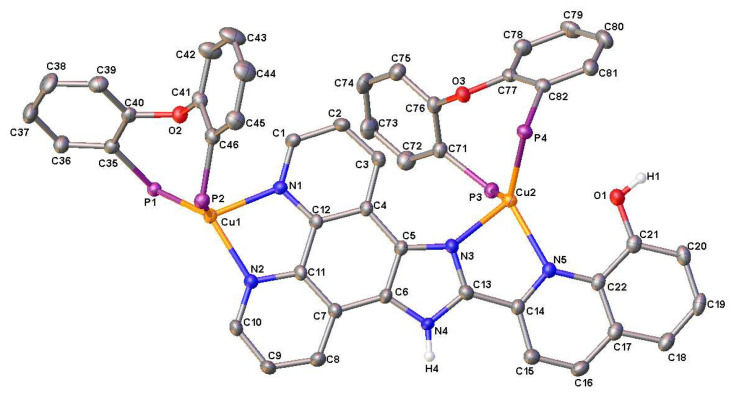
ORTEP drawing of the cation of complex **3** with thermal ellipsoids at 50% probability. For clarity, most of the hydrogen atoms and the benzene rings in the PPh_2_ units of the POP ligand have been omitted.

**Figure 3:**
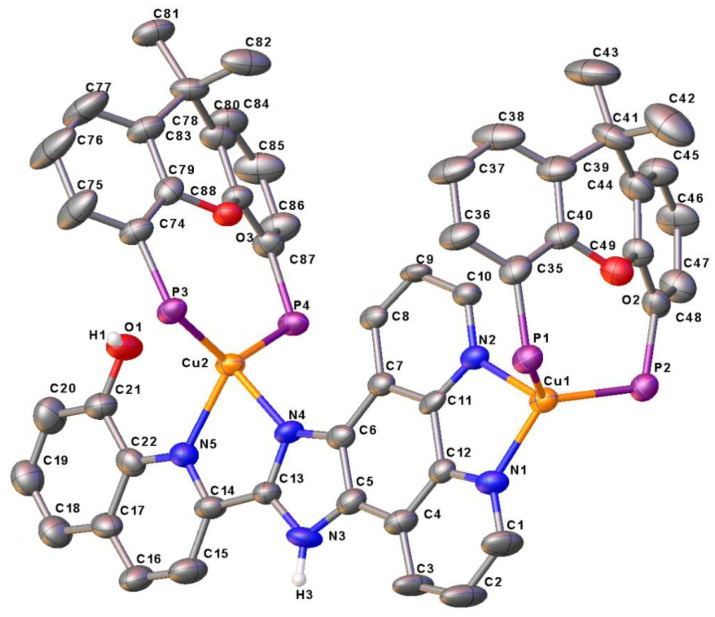
ORTEP drawing of complex **4** with thermal ellipsoids at 50% probability. For clarity, the counter ions, most of the hydrogen atoms and the benzene rings from PPh_2_ units of xantphos are omitted.

The crystallographic data and selected refinement parameters for complexes **1**·CH_2_Cl_2_, **3** and **4** are listed in Table S1 (see Supplementary Materials), while the important bond lengths and bond angles are provided in Table 1. The perspective views of their corresponding cationic structures are shown in Figure 1, Figure 2 and Figure 3.

As shown in Figure 1, complex **1** has a mononuclear structure with two molecules in an asymmetric unit of its crystal structure. The distorted tetrahedral Cu(I) centers are completed by the two nitrogen atoms of the phen unit of ipqH_2_ and two phosphorus atoms from POP. Complexes **3** and **4** both have binuclear core structures, as shown in Figure 2 and Figure 3, with two typical distorted tetrahedral Cu(I) centers completed by the two nitrogen atoms from the phen unit or from imidazole and quinolone rings of the ipqH_2_ ligand and two phosphorus atoms from the diphosphine ligands POP or xantphos. Such structures are similar to reported Cu(I) complexes of 1*H*-imidazo[4,5-*f*][1,10]phenanthroline [13]. The bond lengths of Cu–P are in the range of 2.2281(7)–2.3142(7) Å. The Cu–N distances involving the phen unit in the range of 2.047(5)–2.098(2) Å are shorter than those involving nitrogen atoms of imidazole and quinolone rings in the range of 2.098(2)–2.204(2) Å. The N–Cu–N, N–Cu–P and P–Cu–P bond angles are within the ranges of 79.2(2)–80.78(19)°, 98.62(6)–130.13(6)°, and 112.99(6)–129.85(7)°, respectively, which are comparable to the values reported in the literature [11,12,13,14,15,16,17].

**Figure 4:**
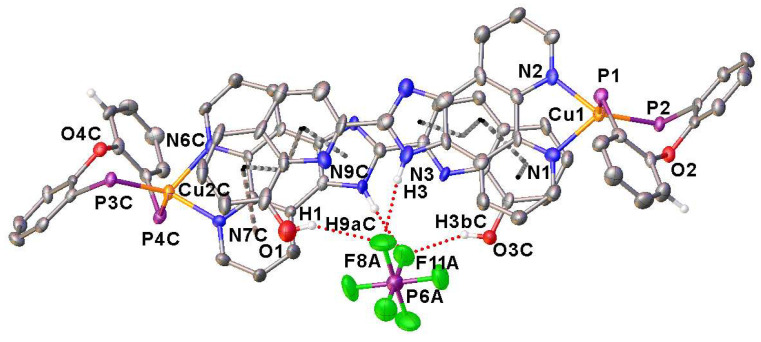
The hydrogen bonds between the cation dimer formed through π⋯π interactions and one PF_6_^−^ ion for complex **1**. For clarity, most of the hydrogen atoms and the benzene rings from PPh_2_ units of POP are omitted.

As shown in Figure 1, strong C–H⋯π interactions between two [Cu(ipqH_2_)(POP)]^+^ cations for complex **1** can be observed, where *d*_H…Cg_ = 2.531 Å and 2.793 Å, *d_Cg_*_…Cg_ = 3.440 Å and 3.706 Å, ∠C–H⋯Cg = 160° and 162°, respectively. Here, Cg represents the center point of the corresponding benzene ring [27]. Intramolecular O–H⋯N hydrogen bonds were observed between –OH and the nitrogen atoms of the quinioline ring (d(O1–H1⋯N5) = 2.704(8) Å, ∠ O1–H1⋯N5 = 113.9°; d(O3–H3B⋯N10) = 2.710(6) Å, ∠ O3–H3⋯N8 = 114.1°) [11,14]. Additionally, as shown in Figure 4, two [Cu(ipqH_2_)(POP)]^+^ cations also form a cationic dimer through eightfold offset π⋯π interactions between the ipqH_2_ ligands (see Table S2) [16], and such a cation dimer further strengthens the connection by the N–H⋯F and O–H⋯F hydrogen bonds with the same hexafluorophosphate ion (see Figure 4 and Table S3) [28,29]. These dimers also further assemble into a zigzag chain via the C–H⋯π interactions mentioned above (see Figure S1). A π⋯π interaction between the ipqH_2_ ligands in the crystal structures of complexes **3** and **4** was not observed. Both N–H⋯F (PF_6_^−^) and O–H⋯F (PF_6_^−^) hydrogen bonds were observed in complex **3**, and only the N–H⋯F (PF_6_^−^) hydrogen bond was observed in complex **4** (see Figures S2 and S3 and Table S3).

The dihedral angles of the quinoline ring and imidazo[1,10]phenanthroline plane are 3.90° and 4.83° in complex **1** and 5.41° and 4.21° in complexes **3** and **4**, respectively. These small dihedral angles indicate that the ligand ipqH_2_ maintains good coplanarity and conjugation in these complexes [11,14,27]. Meanwhile, the similarity of these dihedral angles also indicates that although two N atoms from the quinoline and imidazole rings form a second chelating coordination center with Cu(I) ions, the rigidity of the ligand may be enhanced [13], but its coplanarity has not changed significantly.

### 2.3. FTIR Spectra

FTIR spectra of the free ligand ipqH_2_ and complexes **1**–**4** are shown in Figures S4–S8. According to studies [11,13,14], the absorption of the free ligand at 3061 cm^−1^ is attributed to the C–H stretching vibration of the aromatic ring. The broad, medium-intensity absorption bands observed in the range of 3098–3329 cm^−1^ with discernible peaks are tentatively assigned to N–H (3098, 3142 and 3173 cm^−1^) and O–H (3329 cm^−1^) stretching vibrations resulting from possible intramolecular/intermolecular hydrogen bonds such as N–H⋯N, N–H⋯O, O–H⋯N, and O–H⋯O.

In complexes **1**–**4**, weak broad absorption bands are still observed above 3000 cm^−1^, but no extensive broad bands similar to those of the free ligand are present. The absorptions at 3052–3055 cm^−1^ are assigned to C–H stretching vibrations. N–H stretching vibrations are observed at 3072 cm^−1^ for complex **1**, 3139 and 3077 cm^−1^ for complex **2**, and 3330 cm^−1^ for complex **3**, respectively. For complex **4**, the N–H vibration overlaps with the C–H vibration and is not distinct. The O–H stretching vibrations in complexes **1**–**4** are located at 3362, 3367, 3456, and 3325 cm^−1^, respectively. Additionally, strong absorption peaks observed in the range of 842–843 cm^−1^ correspond to the stretching vibration of the PF_6_^−^ ion, confirming the presence of the counterion PF_6_^−^ in these complexes [14].

### 2.4. ^1^H and ^31^P{^1^H} NMR Spectra

The ^1^H spectra of the ligand ipqH_2_ and Cu(I) complexes **1**–**4** are shown in Figures S9–S13. The ligand ipqH_2_ and complexes **1**–**4** show single peaks characteristic of the –NH group of imidazole rings at 13.99–14.32 ppm [11,13,14,30,31], and phenolic hydroxyl –OH at 9.82–9.85 ppm [26,30,31]. Other signals also match their structures well. The corresponding ^31^P{^1^H} NMR results of complexes **1**–**4** are shown in Figures S14–S17. The mononuclear complexes **1** and **2** exhibit single peaks at −11.59 and −12.83 ppm, respectively. The binuclear complexes **3** and **4** show a set of signals at −11.58 and −12.86 ppm which are similar to those of complexes **1** and **2**, and also close to what has been reported in studies [11,13,14]. The other single peaks at −19.29 and −18.43 ppm for complexes **3** and **4** are assigned to the P atoms of the diphosphine ligands coordinated to Cu2 centers [13]. For complex **4**, weak minor peaks at −14.96 and −20.82 ppm were also observed, likely due to the asymmetry of ipqH_2_ and conformational variations in xantphos [32,33]. All complexes (**1**–**4**) display characteristic septet signals around −144.20 ppm, corresponding to the hexafluorophosphate anion (PF_6_^−^) [14].

### 2.5. Photophysical Properties

#### 2.5.1. Electron Absorption and Emission Spectra in DMSO

**Figure 5:**
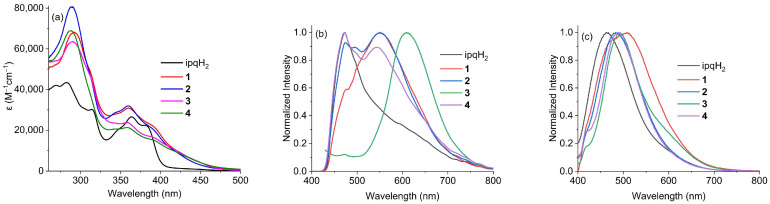
Electron absorption spectra (**a**) and emission spectra in the solid state (**b**) and in DMSO solutions (**c**) for ipqH_2_ and complexes **1**−**4**.

Although the synthesis of ipqH_2_ has been reported in the literature [26], its photophysical properties such as absorption and emission spectra remain unexplored. Here, the UV-vis electron absorption spectra and emission spectra of all compounds in the solid state and in DMSO (2.5 × 10^−5^ mol·L^−1^) solutions at 298 K are shown in Figure 5. The excitation spectra of all compounds in the solid state and in DMSO (2.5 × 10^−5^ mol·L^−1^) solutions at 298 K are depicted in Figure S18. The corresponding data are listed in Table 2. To align with the excitation wavelength of the UV lamp used during photography, the excitation wavelength for obtaining the emission spectra was consistently set at 365 nm, with a filter at 395 nm or 430 nm.

The UV-vis spectrum of the free ligand ipqH_2_ exhibits two main absorption regions. According to the magnitude of the extinction coefficients (*ε*) and the characteristics of the spectral band, the intense higher-energy bands below 330 nm with absorption peaks at 283 and 315 nm (*ε* ≈ 10^4^) are tentatively attributed to ligand-centered (LC) π → π* transitions of the aromatic and heteroatomic components. The push-pull structure of ipqH_2_ is also similar to that of the 2,5-bis(1*H*-imidazo[4,5-*f*][1,10]phenanthrolin-2-yl)benzene-1,4-diol with donor-acceptor units reported by Singh’s group [34], so the broad low-energy bands with moderate intensity at 364 and 381 nm (*ε* ≈ 10^4^) are probably caused by an intra-ligand charge transfer (ILCT) transition, resulting from the push-pull architecture between the electron-donating 8-hydroxyquinoline unit and the electron-accepting 1*H*-imidazo[4,5-*f*][1,10]phenanthroline unit. The weaker tail absorption between 400~500 nm is attributed to n → π* transitions.

For complexes **1**–**4**, the absorption profiles are highly similar (ignoring minor shifts and *ε* variations) and largely independent of the diphosphine ligands, indicating comparable frontier orbitals and electronic transitions. The TD-DFT theoretical calculations for the complex [Cu(nimpH)(POP)]PF_6_ (where nimpH = 2-(2-naphthyl)-1*H*-imidazo[4,5-*f*][1,10]phenanthroline) reveal that its HOMO is primarily contributed by the Cu(I) ion and the POP ligand, with an additional portion originating from the naphthyl ring. In contrast, the LUMO is mainly localized on the 1*H*-imidazo[4,5-*f*][1,10]phenanthroline unit [17]. The electronic transitions in the absorption spectrum thus involve ILCT, metal-to-ligand charge transfer (MLCT), and ligand-to-ligand charge transfer (LLCT) processes. Given the structural similarity between nimpH and ipqH_2_, and considering that the donor-acceptor (D-A) architecture of ipqH_2_ should exhibit enhanced push-pull electronic characteristics upon coordination to the Cu(I) ion, it is reasonable to anticipate the presence of an ILCT transition between the donor and acceptor units in complexes **1**–**4** as well. To further elucidate the nature of the electronic transitions, we compared the experimental absorption spectra of complexes **1**–**4** with those of the free ligands and known Cu(I) complexes [11,13,14,16]. The high-energy bands (*λ* ≤ 315 nm) are tentatively assigned to LC π → π* transitions involving ipqH_2_ and diphosphine ligands. The shoulder absorption near 360 nm and a tail extending to around 500 nm primarily originate from the ILCT of ipqH_2_, possibly accompanied by LLCT between the imine and diphosphine ligands, as well as MLCT from the d(Cu) orbital to the 1*H*-imidazo[4,5-*f*][1,10]phenanthroline unit. Certainly, further theoretical studies are necessary to precisely assign the molecular orbitals involved in the electronic absorption spectra in the future.

In the solid state, the ligand and all complexes exhibited similar emission peaks in the range of 472–477 nm. With reference to the literature [17,34], these emission peaks are tentatively assigned to LC π* → π fluorescence, while emissions in the range of 543–551 nm are preliminarily attributed to intra-ligand charge transfer- and metal-to-ligand charge transfer ((IL+ML)CT)-dominated phosphorescence for complexes **1**, **2** and **4**. The emission peaks observed at about 593 nm for ipqH_2_ are tentatively attributed to ILCT emission. The luminescence at 609 nm for complex **3** results most likely from ^3^LLCT luminescence, since it is the lowest-lying excited state in general [35]. Meanwhile, such ^3^LLCT luminescence may be mixed with some ^3^(IL + ML)CT transition luminescence. Such mixed excited states have also been previously reported in other heteroleptic Cu(I) complexes, based on mixed phosphine/thiocarbamoyl-pyrazoline/halide ligands [36]. In DMSO solution, the luminescence of ligand ipqH_2_ at around 463 nm is also primarily attributed to LC π* → π emission. The emission spectra of the four complexes are similar, with maximum emission wavelengths ranging from 484 to 501 nm, exhibiting a redshift of 21–38 nm compared to the ligand ipqH_2_. Among them, complex **1** shows the largest luminescence redshift. Compared to the absorption spectra and considering their large Stokes shifts, as well as referencing the literature [11,13,14,16], the luminescence of the complexes in DMSO is preliminarily assigned to the mixture of LC emissions and ^3^(IL + ML)CT emissions, and probably also mixed with some contributions from ^3^LLCT, especially for complex **1 [17]**. The emission spectrum of complex **1** in DMSO is relatively broad, with a full width at half maximum of approximately 143 nm, suggesting the possible coupling of dual peaks and the potential existence of more than one excited state.

#### 2.5.2. Solvent Effects on Photoluminescence

**Figure 6:**
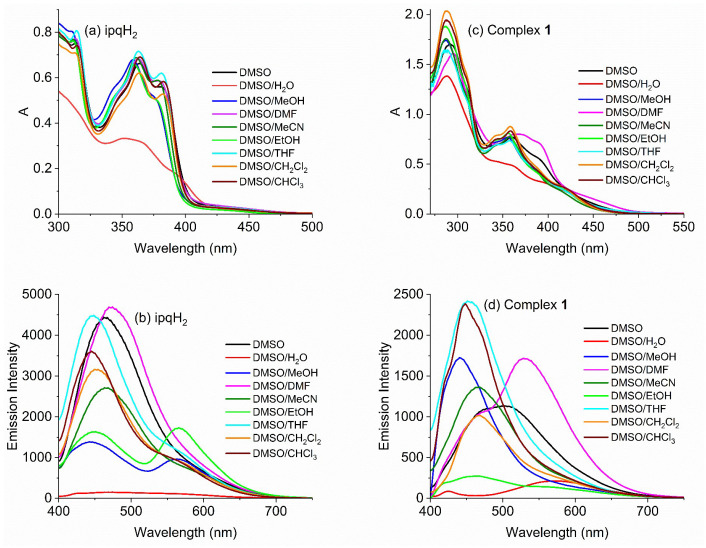
The UV-vis absorption spectra and emission spectra of ligand ipqH_2_ (**a**,**b**) and complex **1** (**c**,**d**) in DMSO-containing mixed solvents (V_DMSO_/V_other solvent_ = 1:9).

**Figure 7:**
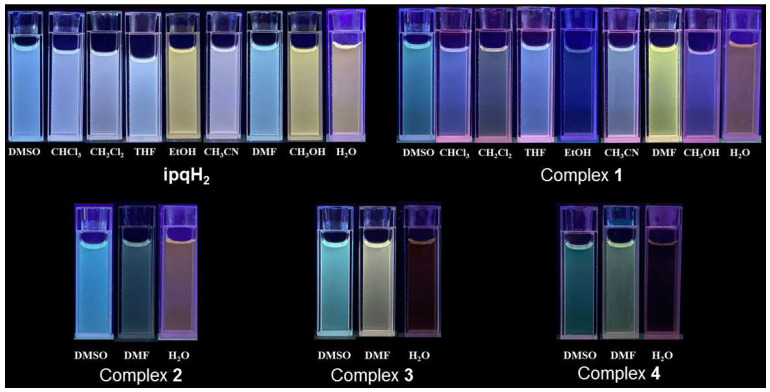
Luminescence photos of ligand ipqH_2_ and complexes **1**−**4** in different mixed solvents (V_DMSO_:Vother solvent = 1:9). The solvents shown in the figure are the second solvents other than DMSO.

To investigate the sensing properties of ligand ipqH_2_ and complexes **1**–**4** toward different solvents, we measured their UV-vis absorption and emission spectra in various solvents of different polarities (including protic and aprotic solvents) (see Figure 6 and Figure S19) and captured luminescence photos under 365 nm UV lamp excitation (with a 395 nm filter) (see Figure 7). During testing and imaging, the concentration of all samples was maintained at 2.5 × 10^−5^ mol·L^−1^. Due to the low solubility of these compounds in common solvents, a DMSO-assisted dissolution method was employed (V_DMSO_:V_other solvent_ = 1:9). The other solvents, listed in order of decreasing polarity, included H_2_O, MeOH, DMF, CH_3_CN, EtOH, THF, CH_2_Cl_2_, and CHCl_3_.

The results showed that, except for the DMSO/H_2_O system, the absorption spectra of ipqH_2_ and complexes **1**–**4** exhibited no significant changes in most solvents compared with those in DMSO. However, ipqH_2_ displayed specific luminescence responses to water, methanol, and ethanol, while complexes **1**–**4** showed significant luminescence responses to water and DMF. Apart from these specific responses, in general, after adding various other solvents to DMSO, the luminescence of ipqH_2_ and the complexes mostly blue-shifted as the polarity of the mixed solvent decreased, indicating that the polarity of their excited states also decreased with reduced solvent polarity [18,19]. Therefore, taking ligand ipqH_2_ and complexes **1** and **2** as examples, we further explored the luminescence mechanisms by investigating the effects of mixed solvents with varying volume fractions of ethanol, water, or DMF on their absorption and emission spectra.

**Figure 8:**
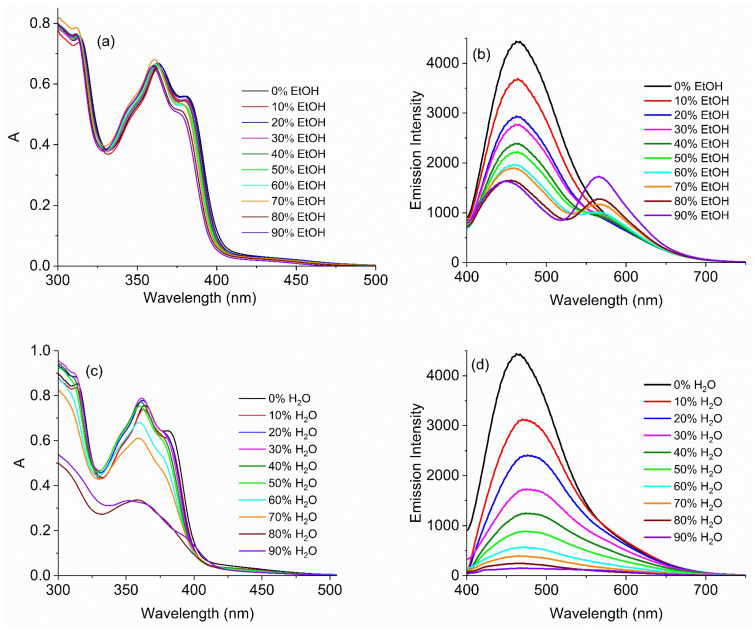
Variation in the UV-vis absorption and emission spectra of ligand ipqH_2_ as a function of EtOH and water fractions (vol%) in DMSO/EtOH (**a**,**b**) and DMSO/H_2_O (**c**,**d**) mixed solvents.

**Alcohol (MeOH/EtOH) solvent effect.** As shown in Figure 8a, as the volume fraction of ethanol (*f*_EtOH_) gradually increased from 0% to 90%, the addition of EtOH only caused a slight decrease in the UV-vis absorption spectra of the ligand ipqH_2_, indicating that no obvious aggregation of ipqH_2_ occurs upon ethanol addition. The spectra also remained relatively stable, without significant red or blue shifts. Although the absorption spectra of ipqH_2_ showed no significant changes, under 365 nm excitation (with a 395 nm filter), as illustrated in Figure 8b, the luminescence of ipqH_2_ gradually decreased and developed a dual-emission feature upon EtOH addition. When *f*_EtOH_ ≥ 60%, this dual-peak characteristic (447 and 561 nm) became increasingly apparent, with a new emission peak emerging near 561 nm that intensified with increasing *f*_EtOH_. A similar trend was observed in DMSO/MeOH mixtures, where dual emissions appeared at 441 and 560 nm (see Figure S20).

**Figure 9:**
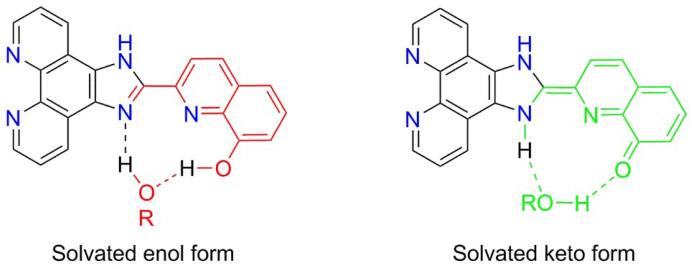
Schematic diagram of possible MeOH- and EtOH-assisted long-range ESIPT excited-state proton transfer mechanisms of ipqH_2_.

Since this phenomenon was observed only in polar protic alcohol-containing solvents, it suggests that the presence of a large amount of MeOH or EtOH disrupted the solvation of ipqH_2_ by DMSO. Meanwhile, these protic alcohol solvents induced an excited-state long-distance proton transfer and isomerization in ipqH_2_, as illustrated in Figure 9, leading to the formation of enol and keto excited-state isomers. Similar solvent-assisted ESPT mechanisms have also been suggested for *o*-hydroxynaphthyl phenanthroimidazole and *o*-hydroxyphenyl phenanthroimidazole [37], 6-32amino-2-(2′-hydroxyphenyl)benzoxazole [38], and 7-hydroxyquinoline [39,40,41]. This resulted in dual emission peaks, shifting the luminescence color of ipqH_2_ from blue to yellow. By comparison with the emissions from the DMSO solution of ipqH_2_ and those in the literature [19,20,21,22,23,39,40,41], the high-energy emission bands at 441 and 447 nm are attributed to the enol-form LC and ILCT excited-state emission, while the low-energy emission bands at 560 and 561 nm are assigned to the keto-form LC and ILCT excited-state emission.

Complexes **1** and **2** showed no significant luminescence response upon the addition of methanol or ethanol. This may be attributed to the potential formation of strong hydrogen bonds between the hexafluorophosphate ions (PF_6_^−^) and the N–H/O–H bonds, as discussed in the crystal structure analysis of complex **1**, which effectively blocked the alcohol-induced excited-state long-range proton transfer and isomerization of the ligand.

In contrast, for the binuclear complexes **3** and **4**, the N atoms on the imidazole C = N double bond and the quinoline N atom were already coordinated to the Cu(I) center, eliminating the possibility of ESPT and isomerization. Thus, they also exhibited no significant luminescence response to methanol or ethanol.

**Figure 10:**
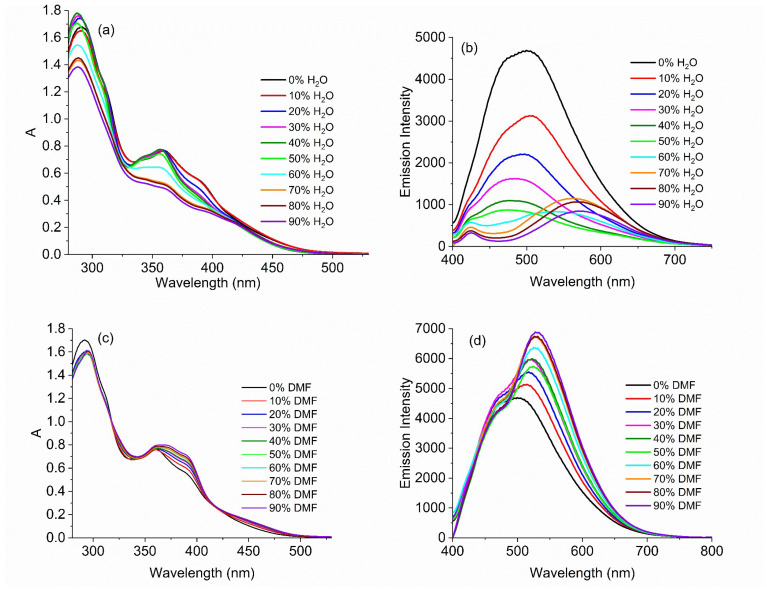
Variation in the UV-vis absorption and emission spectra of complex **1** as a function of H_2_O and DMF fractions (vol%) in DMSO/H_2_O (**a**,**b**) and DMSO/DMF (**c**,**d**) mixed solvents.

**Water effect**. As shown in Figure 10, the absorption spectra of ipqH_2_, complexes **1** and **2** remained largely unchanged when the water volume fraction (*f*_w_) was in the range of 0–50%. However, when *f*_w_ ≥ 60%, the absorption below 400 nm decreased significantly, indicating that the high dielectric constant of water substantially reduced the solubility of ipqH_2_ and the complexes, leading to aggregation, precipitation, or solvation layer disruption, even though the solution remained visibly clear to the naked eye. Additionally, water might protonate the quinoline N or imidazole N, altering the π-conjugation system and affecting the ground-state electronic structure and electronic transitions.

On the other hand, as illustrated in Figure 8, Figure 10 and Figure S21 (excited at 365 nm, with a 395 nm filter), the luminescence intensity of ipqH_2_ and complexes **1** and **2** gradually weakened as *f*_w_ increased from 0% to 90%. For ipqH_2_, the emission maximum wavelength first red-shifted from 463 nm to 476 nm and then blue-shifted back to 465 nm. The luminescence quenching may have been caused by the following possible mechanisms. The high-energy O–H vibrational modes in water enhanced non-radiative decay (non-radiative energy dissipation), reducing luminescence efficiency [14]. At high water fractions (*f*_w_ ≥ 60%), low water solubility triggers molecular aggregation. This may result in aggregation-caused quenching (ACQ), prompted by the similar π···π interactions observed in complex **1** [42]. As a protic solvent, water might induce long-range excited-state proton transfer (similar to that in DMSO/MeOH and DMSO/EtOH), but its dominant effects here are aggregation and solubility changes.

Complexes **1** and **2** show obvious red-shifted emissions with high water content (*f*_w_ ≥ 60%). At *f*_w_ = 90%, the emission maxima of complexes **1** and **2** red-shifted significantly to 569 nm and 574 nm, respectively. This red shift likely arises from competitive hydrogen bonding: water competes with PF_6_^−^ ions for H-bonding sites, forming N(O)–H⋯OH_2_ interactions. This lengthens the O–H bond, stabilizing a highly polar solvent-stabilized (IL + ML)CT state, lowering the excited-state energy level. When excited at 365 nm, the emission colors of ligand ipqH_2_ and complexes **1**–**4** in DMSO/H_2_O (*v*/*v*, 1:9) appeared as yellow, orange, orange, red, and reddish-brown, respectively, as shown in Figure 7.

**DMF effect**. As discussed earlier, the broad emission peak of complex **1** in DMSO suggests the existence of more than one excited state. As the volume fraction of DMF (*f*_DMF_) gradually increases in the DMSO/DMF mixed solvent (see Figure 10), the emission spectrum of complex **1** progressively splits into more distinct shoulder peaks. The peak around 470 nm slightly weakens, while the peak near 501 nm gradually red-shifts to approximately 530 nm and intensifies, accompanied by a noticeable change in the solution’s luminescence color from blue to bright yellow.

**Figure 11:**
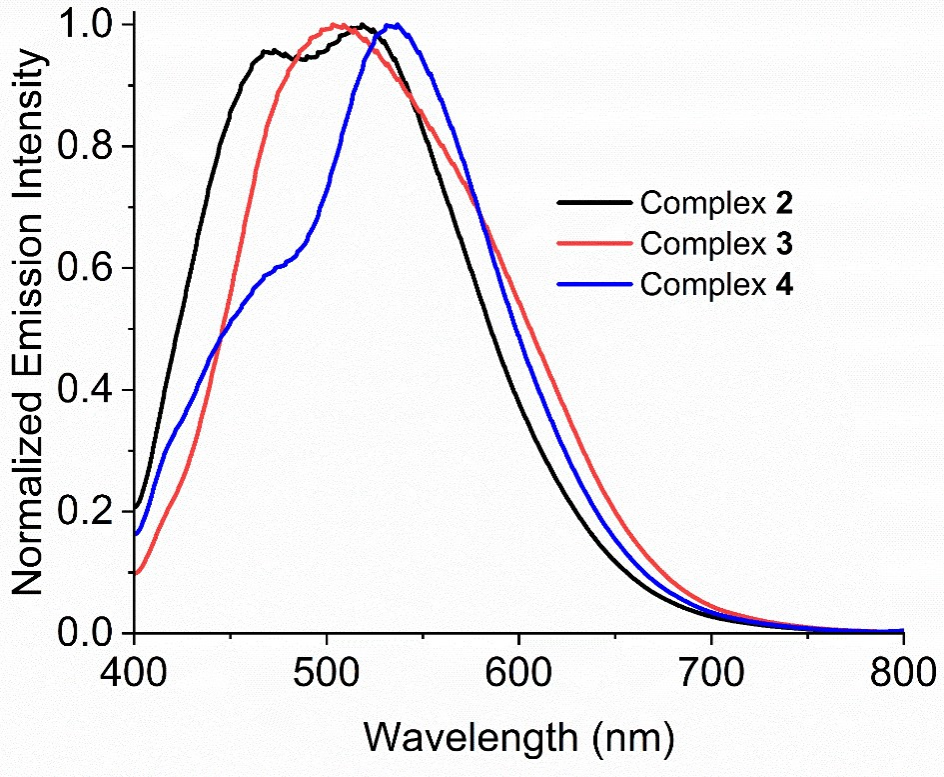
Emission spectra of complexes **2**–**4** in DMF.

In contrast to complex **1**, the luminescent response of complex **2** to DMF addition during the testing period was the opposite (see Figure S21). Instead of further peak splitting and red shift, its emission peak blue-shifted from 484 nm in DMSO to 468 nm (a 16 nm blue shift), with an approximately 56% increase in emission intensity. However, in pure DMF, the emission peak of complex **2** split, with the first peak (maximum at 468 nm) remaining largely unchanged, while the second peak red-shifted to 518 nm (see Figure 11). Some reports attributed such luminescent responses to the coordination of DMF with the metal center [43]. We conducted mass spectrometry tests on complexes **1** and **2** in DMSO/DMF (*v*:*v*, 1:9), but found no signal peaks indicating DMF participation in coordination, preliminarily ruling out the possibility of DMF coordinating with the Cu(I) ions. Therefore, we speculate that the response of the complexes to DMF is closely related to PF_6_^−^ and the diphosphine ligands.

In complex **1**, the PF_6_^−^ ion forms N−H⋯F and O−H⋯F hydrogen bonds with the ipqH_2_ ligand, enhancing the rigidity of ipqH_2_. In the presence of a large amount of DMSO, DMSO may displace the PF_6_^−^ ion and form intermolecular hydrogen bonds with the ligand ipqH_2_. Upon the addition of DMF, which also acts as a stronger hydrogen bond acceptor, DMF competes to form hydrogen bonds with ipqH_2_, weakening the interactions among PF_6_^−^ ion, DMSO, and the ipqH_2_ ligand. This exposes the excited state of complex **1** to a highly polar solvent environment while accelerating hydrogen bond reorganization kinetics, resulting in a red shift of the (IL + ML)CT emission spectrum.

The differences in luminescent behavior between complexes **2** and **1** in DMSO/DMF may arise from structural variations in the bidentate diphosphine ligands, xantphos and POP, leading to differing solvent effects of DMF on the complexes. The lower steric hindrance and flexible ether linkage of the POP ligand allow significant distortion and multiple conformations in the excited state of complex **1**. DMF stabilizes these distorted configurations through hydrogen bonding, causing the splitting of dual peaks and the redshift of the (IL + ML)CT peak. In contrast, the rigid framework and high steric hindrance of the xantphos ligand largely shield the metal center from short-term interference by DMF in the DMSO/DMF solvent and protect the original hydrogen bonds, maintaining the relative stability of the excited state, unless disturbed by high concentrations of DMF (e.g., pure DMF).

Both the DMSO solution and the DMSO/DMF (*v*:*v* = 1:9) solution of complex **3** exhibit broad emission spectra, with the maximum emission peak appearing at 494 nm. However, the emission spectrum in DMSO/DMF (*v*:*v* = 1:9) is broader. In pure DMF, the emission peak of complex **3** further broadens (see Figure 11), and the maximum red-shifts to 503 nm. For complex **4**, the emission spectrum in DMSO/DMF (*v*:*v* = 1:9) becomes significantly broader compared to that in DMSO (maximum emission at 490 nm), with a flattened peak top appearing around 494–499 nm. In pure DMF (see Figure 11), the emission spectrum of complex **4** further broadens and splits into two peaks, with maxima at 470 nm and 537 nm, exhibiting behavior similar to that of complex **1** in DMSO/DMF. The response of complexes **3** and **4** to DMF in mixed solvents is also attributed to DMF modulating the luminescence of the Cu(I) complexes through hydrogen-bond competition and solvation shell reorganization.

The high-energy emission peaks of complexes **1**–**4** in DMSO/DMF and DMF closely match the emission position of the ipqH_2_ ligand, and are therefore assigned to LC transitions of the ligand perturbed by the metal center. Meanwhile, their low-energy emission peaks in the range of 503–537 nm are primarily attributed to ^3^(IL + ML)CT transitions.

## 3. Materials and Methods

### 3.1. Chemicals and Instrumentation

The 5,6-Diamino-1,10-phenanthroline (dap) was purchased from Alpha Chemical Co., Ltd., Zhengzhou, China. The 8-Hydroxyquinoline-2-carbaldehyde was obtained from Beijing Innochem Science & Technology Co., Ltd., Beijing, China. Xantphos and POP were both sourced from Shanghai J&K Chemical Technology Co., Ltd., Shanghai, China. The copper salt [Cu(CH_3_CN)_4_]PF_6_ was acquired from Shanghai Bide Pharmatech Ltd., Shanghia, China. All of the above chemicals were of analytical grade and used as received without further purification.

The solvents used in the experiments, including anhydrous ethanol (EtOH), *n*-hexane, methanol (CH_3_OH), dichloromethane (CH_2_Cl_2_), dimethyl sulfoxide (DMSO), acetonitrile (CH_3_CN), chloroform (CHCl_3_), N,N-dimethylformamide (DMF), and tetrahydrofuran (THF), were all purchased from Xilong Scientific Co., Ltd., Guangzhou, China and were of analytical grade.

Nuclear magnetic resonance (NMR) spectroscopy was conducted on a Bruker-400 MHz NMR spectrometer. The ^1^H NMR spectra were measured using DMSO-*d*_6_ as the solvent and Me_4_Si as the internal standard. The ^31^P {^1^H} NMR measurement procedure was as follows. First, the sample was dissolved in DMSO-*d*_6_, and the deuterium signal was used to activate the lock function to dynamically stabilize the magnetic field strength. After optimizing magnetic field homogeneity through shimming, the instrument configured the ^31^P channel parameters (spectral width, pulse width, etc.) and indirectly calibrated the chemical shift based on a preset reference frequency associated with the deuterium lock (e.g., the ^31^P chemical shift of H_3_PO_4_ was set as 0 ppm). Following the acquisition of the free induction decay (FID) signal, Fourier transformation and data processing directly yielded the calibrated ^31^P NMR spectrum without the need for additional internal or external standards. High-resolution mass spectrometry (HRMS) was performed on an AB SCIEX 4600 Triple TOF MS instrument in positive ion mode, with CH_2_Cl_2_/CH_3_OH as the mobile phase (see Figures S22–S26). Infrared (IR) spectroscopy was carried out using the KBr pellet method on a Bruker Optics TENSOR 27 FT-IR spectrometer. Ultraviolet–visible (UV–vis) absorption and fluorescence emission spectra were measured on a UV–vis spectrophotometer (UV-1800PC) and a Hitachi fluorescence spectrometer (F-4600), respectively.

The X-ray single-crystal diffraction (SCD) test was performed on an X-ray single-crystal diffractometer (Bruker Smart APEX II). All crystals were grown in test tubes containing a mixed solvent of CH_2_Cl_2_/*n*-hexane. To prevent crystal desiccation, the testing process was conducted at 150 K under a stream of liquid nitrogen. High-quality crystals were selected, immediately coated with colorless nail polish, and then transferred to an X-ray single-crystal diffractometer for measurement.

Data integration and intensity correction were performed using the Bruker SAINT program, while absorption correction was carried out with the SADABS program reported in 2009 [44]. The crystal structures were solved by direct methods using Olex2 and subsequently refined anisotropically for all non-hydrogen atoms using the SHELXT and SHELXL program reported in 2015 [45,46]. All the hydrogen atoms, including those from N–H and O–H groups, were generated theoretically according to the idealized geometric parameters and refined isotropically by riding on their corresponding parent atoms. Some severely disordered solvent molecules were not resolved. The contribution of some unresolved solvent molecules and some resolved but disordered solvent molecules in complexes **1** (one dichloromethane and 0.75 water molecules), **3** (one dichloromethane molecule and two water molecules) and **4** (five dichloromethane molecules and some unresolved solvent molecules) to the structure factors was removed using the SQUEEZE routine in the program PLATON reported in 2015. The final cycles of refinement were performed on the squeezed.hkl data [47].

### 3.2. Synthesis

#### 3.2.1. IpqH_2_

The ligand ipqH_2_ was synthesized following a procedure similar to that reported in the literature [26], with slight modifications in the workup process. A mixture of 8-hydroxyquinoline-2-carbaldehyde (173.2 mg, 1.0 mmol) and dap (210.2 mg, 1.0 mmol) in 100 mL of ethanol was refluxed at 82 °C for 25 h, yielding a clear red solution. After cooling to room temperature, the solution was concentrated by evaporation to approximately 5 mL, followed by the addition of 150 mL of n-hexane and sonication, which rapidly produced a yellowish-brown precipitate. The precipitate was collected by filtration, washed three times with n-hexane, and dried to afford the target product as a yellowish-brown solid. Yield: 86.9% (631.4 mg). ^1^H NMR (400 MHz, DMSO-*d*_6_, *δ*, ppm): 13.99 (s, 1H, NH), 9.83 (s, 1H, OH), 9.10 (dd, J = 13.6 Hz, J′ = 2.8 Hz, 2H), 8.99 (d, J = 8.0 Hz, 1H), 8.89 (d, J = 8.0 Hz, 1H), 8.58 (d, J = 8.8 Hz, 1H), 8.50 (d, J = 8.8 Hz, 1H), 7.95 (dd, J = 8.0 Hz, J′ = 4.4 Hz, 1H), 7.88 (dd, J = 7.6 Hz, J′ = 4.4 Hz, 1H), 7.58–7.52 (m, 2H), 7.26 (d, J = 6.8 Hz, 1H). HRMS (*m*/*z*): 364.1176 [M + H^+^] (calcd. 364.1193). Characteristic IR spectra (KBr, cm^−1^): 3329 m (O–H), 3173 m (N–H), 3142 m (N–H), 3097 m (N–H).

#### 3.2.2. [Cu(ipqH_2_)(POP)]PF_6_ (**1**)

Under a dry argon stream at room temperature in a Schlenk flask, [Cu(CH_3_CN)_4_]PF_6_ (37.3 mg, 0.1 mmol), ipqH_2_ (36.3 mg, 0.1 mmol), and POP (53.9 mg, 0.1 mmol) were added to 30 mL of CH_2_Cl_2_. The mixture was stirred at room temperature for 5 h, followed by vacuum filtration to remove trace precipitates. The resulting clear filtrate was transferred to a test tube, and *n*-hexane was slowly layered along the tube wall for crystal growth via solvent diffusion. The sealed system was stored in the dark for one week, yielding yellow prismatic crystals. The crystals were prone to efflorescence; therefore, the samples were typically dried under an infrared lamp prior to yield calculation. Yield: 64.7% (71.8 mg). ^1^H NMR (400 MHz, DMSO-*d*_6_, *δ*, ppm): 14.30 (s, 1H, N*H*), 9.83 (s, 1H, O*H*), 9.12 (d, *J* = 8.0 Hz, 1H), 9.03 (d, *J* = 8.4 Hz, 1H), 8.89 (s, br, 2H), 8.59 (d, *J* = 8.4 Hz, 1H), 8.52 (d, *J* = 8.8 Hz, 1H), 8.02–7.92 (m, 2H), 7.59–7.53 (m, 2H), 7.46 (t, *J* = 8.0 Hz, 2H), 7.33–7.19 (m, 15H), 7.12 (t, *J* = 7.6 Hz, 2H), 7.01–7.00 (m, 8H), 6.69 (s, br, 2H). ^31^P {^1^H} NMR (162 MHz, DMSO-*d*_6_, *δ*, ppm): −11.59 (s), −144.20 (septet, PF_6_^−^). HRMS (*m*/*z*): 964.2038 [Cu(ipqH_2_)(POP)]^+^ (calcd. 964.2032). Characteristic IR spectra (KBr, cm^−1^): 3362 m (O–H), 3072 m (N–H), 843 vs (PF_6_^−^).

#### 3.2.3. [Cu(ipqH_2_)(xantphos)]PF_6_ (**2**)

The synthesis and drying of complex **2** followed a procedure similar to that of complex **1**, with the only difference being the replacement of the diphosphine ligand with xantphos (57.9 mg, 0.1 mmol). After allowing the mixture to stand for two weeks, orange-yellow polyhedral crystals were obtained. Yield: 39.8% (45.8 mg). ^1^H NMR (400 MHz, DMSO-*d*_6_, *δ*, ppm): 14.29 (s, 1H, N*H*), 9.82 (s, 1H, O*H*), 9.07 (s, br, 2H), 8.61–8.50 (m, 4H), 7.97 (s, br, 2H), 7.90 (d, *J* = 7.6 Hz, 2H), 7.60–7.54 (m, 2H), 7.32–7.23 (m, 7H), 7.12 (t, *J* = 7.6 Hz, 8H), 6.95–6.94 (m, 8H), 6.61–6.60 (m, 2H), 1.77 (s, 6H). ^31^P {^1^H} NMR (162 MHz, DMSO-*d*_6_, *δ*, ppm): −12.83 (s), −144.19 (septet, PF_6_^−^). HRMS (*m*/*z*): 1004.2374 [Cu(ipqH_2_)(xantphos)]^+^ (calcd. 1004.2344). Characteristic IR spectra (KBr, cm^−1^): 3367 w (O–H), 3139 w (N–H), 3077 w (N–H), 843 vs (PF_6_^−^).

#### 3.2.4. [Cu_2_(ipqH_2_)(POP)_2_](PF_6_)_2_ (**3**)

Under a dry argon atmosphere at room temperature in a Schlenk flask, [Cu(CH_3_CN)_4_]PF_6_ (37.3 mg, 0.1 mmol), ipqH_2_ (18.2 mg, 0.05 mmol), and POP (53.9 mg, 0.1 mmol) were dissolved in 30 mL of CH_2_Cl_2_. The mixture was stirred at room temperature for 4 h, followed by vacuum filtration to remove trace precipitates. The resulting clear filtrate was transferred to a test tube, and *n*-hexane was slowly layered along the tube wall for crystal growth via solvent diffusion. After sealing the system and storing it in the dark for approximately six days, brown-yellow block-shaped crystals were obtained. The samples were also dried under an infrared lamp prior to yield calculation due to the efflorescent nature of the crystals. Yield: 67.3% (62.5 mg). ^1^H NMR (400 MHz, DMSO-*d*_6_, *δ*, ppm): 14.31 (s, 1H, N*H*), 9.84 (s, 1H, O*H*), 9.12–9.03 (m, 2H), 8.89 (s, br, 2H), 8.61 (d, *J* = 8.0 Hz, 1H), 8.52 (d, *J* = 7.6 Hz, 1H), 8.03–7.94 (m, 2H), 7.60–7.00 (m, 55H), 6.68 (s, br, 4H). ^31^P {^1^H} NMR (162 MHz, DMSO-*d*_6_, *δ*, ppm): −11.58 (s), −19.29 (s), −144.20 (septet, PF_6_^−^). HRMS (*m*/*z*): 1566.2853 {[Cu_2_(ipqH_2_)(POP)_2_]^2+^ − H^+^}^+^ (calcd. 1566.2878). Characteristic IR spectra (KBr, cm^−1^): 3456 w (O–H), 3330 m (N–H), 842 vs (PF_6_^−^).

#### 3.2.5. [Cu_2_(ipqH_2_)(xantphos)_2_](PF_6_)_2_ (**4**)

The synthesis and drying of complex **4** followed a procedure analogous to that of complex **3**, with the sole modification being the substitution of the diphosphine ligand with xantphos (57.9 mg, 0.1 mmol). Through solvent diffusion over eight days, brown-yellow prismatic crystals were obtained. Yield: 49.1% (47.6 mg). ^1^H NMR (400 MHz, DMSO-*d*_6_, *δ*, ppm): 14.32 (s, 1H, N*H*), 9.85 (s, 1H, O*H*), 9.12 (d, *J* = 6.8 Hz, 1H), 9.04 (d, *J* = 7.8 Hz, 1H), 8.63–8.51 (m, 4H), 8.02–7.99 (m, 1H), 7.94–7.89 (m, 3H), 7.76 (d, *J* = 7.6 Hz, 2H), 7.61–7.55 (m, 2H), 7.43–7.23 (m, 29H), 7.12 (t, *J* = 7.2 Hz, 8H), 6.94 (s, br, 8H), 6.64–6.60 (m, 4H), 1.77 (s, 6H), 1.61 (s, 6H). ^31^P {^1^H} NMR (162 MHz, DMSO-*d*_6_, *δ*, ppm): −12.86 (s), −14.96 (s), −18.43 (s), −20.82 (s), −144.20 (septet, PF_6_^−^). HRMS (*m*/*z*): 1646.3608 {[Cu_2_(ipqH_2_)(xantphos)_2_]^2+^ − H^+^}^+^ (calcd. 1646.3507). Characteristic IR spectra (KBr, cm^−1^): 3325 w (O–H), 842 vs (PF_6_^−^).

## 4. Conclusions

This study successfully synthesized and characterized two mono- and two binuclear Cu(I) complexes using an 8-hydroxyquinoline-modified 1*H*-imidazo[4,5-*f*][1,10]phenanthroline ligand. Key findings revealed distinct solvent-mediated excited-state behaviors. In alcoholic solvents, free ipqH_2_ undergoes ESPT-driven enol-keto tautomerism, producing dual emission, but this process is suppressed upon Cu(I) coordination or by hydrogen bonds between ipqH_2_ and PF_6_^−^ anions. Water induces aggregation-caused quenching and vibrational quenching, while also causing an unusual red-shifted emission (~570 nm) in complexes **1** and **2** due to hydrogen bonding stabilization of low-energy (IL + ML)CT states. In DMF, flexible POP-based complexes **1** and **3** exhibit peak splitting and red shift (~30 nm) as the solvent disrupts hydrogen bond networks, whereas rigid xantphos-based complexes **2** and **4** show resistance to short-term solvation effects. The diphosphine ligand’s steric flexibility and PF_6_^−^ anions critically modulate solvent responses. These results establish structure–property relationships for solvent-responsive luminescence in Cu(I) systems, enabling the design of ESPT-based bioprobes, water/DMF-sensitive chemical sensors, and ratiometric solvent polarity indicators. This work advances the understanding of solvent-responsive Cu(I) photophysics and provides key principles for developing optoelectronic materials.

## Figures and Tables

**Table 1:** Selected bond lengths (Å) and angles (º) for **1**·CH_2_Cl_2_, **3** and **4**.

1·CH_2_Cl_2_		3		4	
Cu1–N1	2.067 (5)	Cu1–N1	2.063 (2)	Cu1–N1	2.047 (5)
Cu1–N2	2.092 (5)	Cu1–N2	2.098 (2)	Cu1–N2	2.083 (5)
Cu2–N6	2.068 (5)	Cu2–N3	2.098 (2)	Cu2–N4	2.099 (5)
Cu2–N7	2.069 (5)	Cu2–N5	2.204 (2)	Cu2–N5	2.123 (5)
Cu1–P1	2.2990 (16)	Cu1–P1	2.2281 (7)	Cu1–P1	2.239 (2)
Cu1–P2	2.2306 (16)	Cu1–P2	2.2740 (7)	Cu1–P2	2.2334 (19)
Cu2–P3	2.2395 (16)	Cu2–P3	2.3142 (7)	Cu2–P3	2.2816 (19)
Cu2–P4	2.2948 (16)	Cu2–P4	2.2516 (7)	Cu2–P4	2.2765 (18)
C13–N3	1.351 (8)	C13–N3	1.322 (3)	C13–N3	1.394 (9)
C13–N4	1.321 (8)	C13–N4	1.360 (3)	C13–N4	1.330 (8)
C71–N8	1.323 (7)	C21–O1	1.355 (3)	C21–O1	1.337 (8)
C71–N9	1.363 (7)				
C21–O1	1.389 (8)				
C79–O3	1.361 (7)				
N1–Cu1–N2	80.0 (2)	N1–Cu1–N2	80.35 (8)	N1–Cu1–N2	80.6 (2)
N6–Cu2–N7	80.78 (19)	N3–Cu2–N5	79.36 (8)	N4–Cu2–N5	79.2 (2)
P1–Cu1–P2	114.74 (6)	P1–Cu1–P2	120.80 (3)	P1–Cu1–P2	116.75 (7)
P3–Cu2–P4	112.99 (6)	P3–Cu2–P4	117.20 (3)	P3–Cu2–P4	129.85 (7)
N1–Cu1–P1	99.82 (12)	N1–Cu1–P1	115.00 (6)	N1–Cu1–P1	117.69 (16)
N1–Cu1–P2	124.61 (15)	N1–Cu1–P2	98.62 (6)	N1–Cu1–P2	117.66 (17)
N2–Cu1–P1	99.82 (12)	N2–Cu1–P1	130.13 (6)	N2–Cu1–P1	107.73 (15)
N2–Cu1–P2	128.96 (14)	N2–Cu1–P2	101.31 (6)	N2–Cu1–P2	109.16 (15)
N6–Cu2–P3	118.40 (13)	N3–Cu2–P3	101.51 (6)	N4–Cu2–P3	104.70 (16)
N6–Cu2–P4	105.34 (13)	N3–Cu2–P4	118.24 (6)	N4–Cu2–P4	106.90 (16)
N7–Cu2–P3	127.80 (13)	N5–Cu2–P3	109.26 (6)	N5–Cu2–P3	110.80 (16)
N7–Cu2–P4	106.14 (13)	N5–Cu2–P4	123.61 (6)	N5–Cu2–P4	112.65 (16)

**Table 2:** Photophysical properties of ligand ipqH_2_ and complexes **1**−**4**.

Compound	*λ_abs_*/nm (*ε*/M^−1^cm^−1^) (DMSO)	*λ*_em_/nm (Solid)	*λ*_em_/nm (DMSO)
ipqH_2_	283 (43,370), 315 (30,030), 364 (26,480), 381 (22,400)	473	463
**1**	291 (67,880), 360 (30,830), 400~500	477, 551	501
**2**	291 (80,220), 360 (31,900), 400~500	475, 550	484
**3**	290 (63,530), 360 (23,550), 400~500	472, 609	494
**4**	287 (68,770), 360 (21,160), 400~500	472, 543	490

## Data Availability

Data are supported within the article and Supplementary Materials.

## Supplementary Materials

The following supporting information can be downloaded at https://www.mdpi.com/article/10.3390/molecules30193973/s1, and CCDC 2483205–2483207 contain the supplementary crystallographic data for **4**, **3** and **1**·CH_2_Cl_2_, respectively. These data can be obtained free of charge from the Cambridge Crystallographic Data Centre, 12 Union Road, Cambridge CB2 1EZ, UK; fax: (+44) 1223-336-033; or e-mail: deposit@ccdc.cam.ac.uk. Molecular structures refinement data and X-Ray crystallographic files in CIF format for determination of the three structures are given in the supporting information. Tables and Figures giving additional crystallographic data, select refinement details and photophysical results are provided in Tables S1–S3 and Figures S1–S26.
