# Supplementary material for: Solvent-Responsive Luminescence of an 8-Hydroxyquinoline-Modified 1H-Imidazo[4,5-f][1,10]phenanthroline Ligand and Its Cu(I) Complexes: Excited-State Mechanisms and Structural Effects
Source: Molecules. 2025 Oct 3;30(19):3973. doi: 10.3390/molecules30193973 (PMC12525910; doi:10.3390/molecules30193973)
Supplement: Supplementary file 1 [file molecules-30-03973-s001.zip › molecules-3873064-supplementary.pdf]

Solvent-Responsive Luminescence of an 8-Hydroxyquinoline-Modified 1H-Imidazo[4,5-f][1,10]phenanthroline Ligand and Its Cu(I) Complexes: Excited-State Mechanisms and Structural Effects

Zhenqin Zhao †, Siyuan Liu †, Shu Cui, Yichi Zhang, Ziqi Jiang and Xiuling Li *

School of Chemistry and Materials Science, Jiangsu Normal University, Xuzhou 221116, China

Table S1. Crystallographic data and select refinement details for complexes 1·CH_2Cl_2, 3 and 4

	**1**·CH_2Cl_2 (CCDC	**3** (CCDC 2483206)	**4** (CCDC 2483205)
Empirical formula	$C_{59}H_{43}Cl_2CuF_6N_5O_2P_3$	$C_{94}H_{69}Cu_2F_{12}N_5O_3P_6$	$C_{100}H_{77}Cu_2F_{12}N_5O_3P_6$
Formula weight	1195.33	1857.44	1937.56
Wavelength (Å)	0.71073	0.71073	0.71073
T (K)	150(2)	150(2)	150(2)
Crystal system	monoclinic	monoclinic	triclinic
Space group	$P2_1/c$	$P2_1/n$	P-1
Unit cell dimensions			
a (Å)	12.3147(2)	13.8348(3)	14.5064(4)
b (Å)	46.2327(7)	22.5694(5)	20.5358(6)
c (Å)	20.5615(3)	28.2355(6)	21.1489(6)
α (°)	90.00	90.00	72.957(2)
β (°)	97.8130(10)	91.2750(10)	87.855(2)
γ (°)	90.00	90.00	70.273(2)
V (Å^3)	11597.8(3)	8814.2(3)	5656.8(3)
Z	8	4	2
D_{calc} (Mg·m^{-3})	1.369	1.400	1.138
F(000)	4880	3792	1984
μ (mm^{-1})	0.617	0.670	0.524
Reflections collected	112732	70123	70124
Independent reflections	11602	15939	16272
Data/restraints/parameters	11602/0/1407	15939/0/1099	16272/0/1116
Goodness-of-fit (GOF) on	1.012	1.016	1.036
Reflections with $I > 2\sigma(I)$	7918	12759	12682
Final R indices [$I > 2\sigma(I)$]	0.0504	0.0382	0.0890
wR_2 [$I > 2\sigma(I)$]	0.0997	0.0898	0.2222

R indices (all data)	0.0903	0.0530	0.1072
wR2 (all data)	0.1139	0.0969	0.2355

Table S2. The π···π interactions in the crystal structures of complexes**1**·CH_2Cl_2.

compound	CgI···CgJ	$d_{(Cg\cdots Cg)}$/Å	CgI_Perp/ Å	CgJ_Perp/Å	slippage/Å	dihedral angle/°
**1**·CH_2Cl_2	Cg1···Cg2 (1+*x*, *y*, −1+*z*)	3.470(3)	3.369(2)	3.353(2)	0.892	1.5(3)
	Cg1···Cg3 (1+*x*, *y*, −1+*z*)	3.652(3)	3.374(2)	3.421(2)	1.278	2.0(3)
	Cg4···Cg5 (1+*x*, *y*, −1+*z*)	3.735(3)	3.439(3)	3.468(2)	1.386	2.3(3)
	Cg4···Cg3 (1+*x*, *y*, −1+*z*)	3.629(3)	3.456(3)	3.434(2)	1.174	2.7(3)
	Cg6···Cg7 (−1+*x*, *y*, 1+*z*)	3.504(3)	3.414(2)	3.390(2)	0.886	3.2(3)
	Cg6···Cg8 (−1+*x*, *y*, 1+*z*)	3.656(3)	3.427(2)	3.403(2)	1.336	1.8(3)
	Cg9···Cg10 (−1+*x*, *y*, 1+*z*)	3.729(3)	3.368(2)	3.476(2)	1.349	4.2(3)
	Cg9···Cg8 (−1+*x*, *y*, 1+*z*)	3.608(3)	3.397(2)	3.399(2)	1.211	1.2(3)

Cg1 consists of C14, C15, C16, C17, C22 and N5; Cg2 consists of N8, N9, C63, C64 and C71; Cg3 consists of C62, C63, C64, C65, C69 and C70; Cg4 consists of C17–C22; Cg5 consists of C65–C69 and N7; Cg6 consists of C72, C73, C74, C75, C80 and N10; Cg7 consists of N3, C5, C6, N4 and C13; Cg8 consists of C4, C5, C6, C7, C11 and C12; Cg9 consists of C75–C80; Cg10 consists of N1, C1, C2, C3, C4 and C12 for **1**·CH_2Cl_2.

Table S3. Selected hydrogen bonds in complexes **1**·CH_2Cl_2**, 3** and **4**

compound	D–H/Å	H···A/Å	D···A/Å	∠DHA/(°)	D–H···A
**1**·CH_2Cl_2	0.84	2.25	2.704(8)	114	O1–H1···N5
	0.84	2.26	2.710(6)	114	O3–H3B···N10
	0.84	2.43	3.197(6)	152	O1–H1···F8 (1+*x*, *y*, *z*)
	0.88	2.15	3.022(7)	172	N3–H3···F8 (1+*x*, *y*, *z*)
	0.84	2.16	2.931(6)	152	O3–H3B···F11 (*x*, *y*, 1+*z*)
	0.88	2.22	3.078(6)	166	N9–H9A···F11 (*x*, *y*, 1+*z*)
**3**	0.84	1.90	2.737(3)	172.3	O1–H1···F9 (2–*x*, −1–*y*, 1–*z*)
	0.88	2.41	3.187(3)	148.1	N4–H4···F4 (1/2 + *x*, −1/2 – *y*, 1/2 + *z*)
	0.88	2.10	2.915(3)	152.9	N4–H4···F5 (1/2 + *x*, −1/2 – *y*, 1/2 + *z*)

**4**	0.86	2.05	2.903(9)	169.5	N3–H3···F1

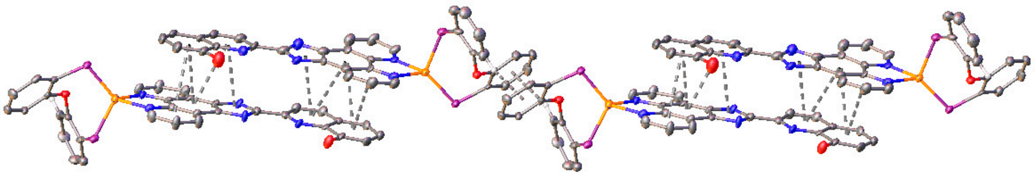

Figure S1. The zigzag chain formed through intermolecular π···π and C–H···π interactions of complex **1**. For clarity, most of the hydrogen atoms and the benzene rings in the PPh_2 units of the POP ligand have been omitted.

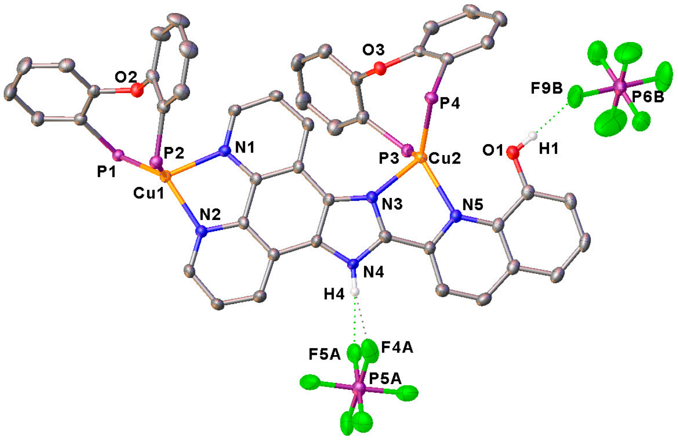

Figure S2. The hydrogen bonds between the cation of complex **3** and PF_6^- ions. For clarity, most of the hydrogen atoms and the benzene rings in the PPh_2 units of the POP ligand have been omitted. Symmetrical codes for A: $1/2 + x$, $-1/2 - y$, $1/2 + z$, B: $2 - x$, $-1 - y$, $1 - z$.

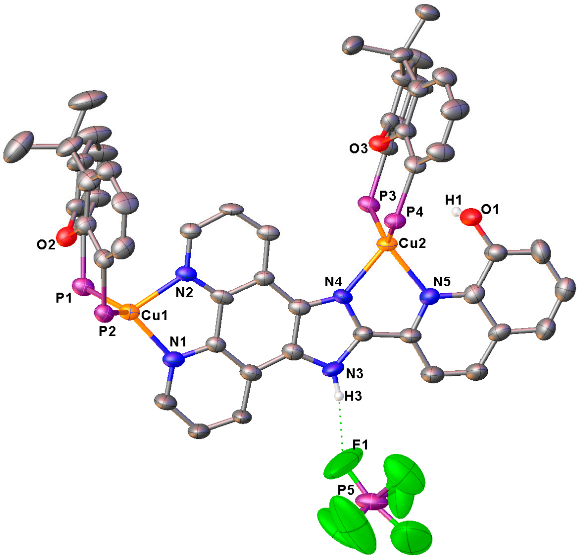

Figure S3. The hydrogen bonds between the cation of complex **4** and PF_6^- ions. For clarity, most of the hydrogen atoms and the benzene rings in the PPh_2 units of the xantphos ligand have been omitted.

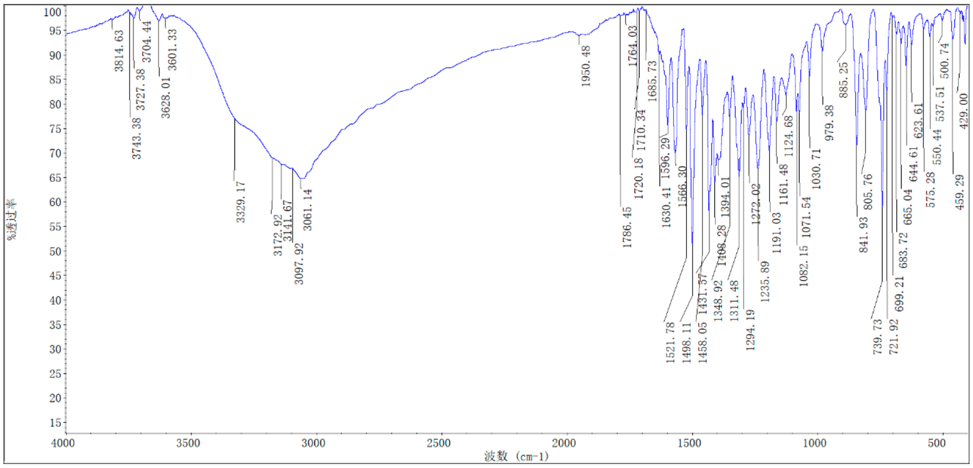

Figure S4. IR spectrum of $ipqH_2$

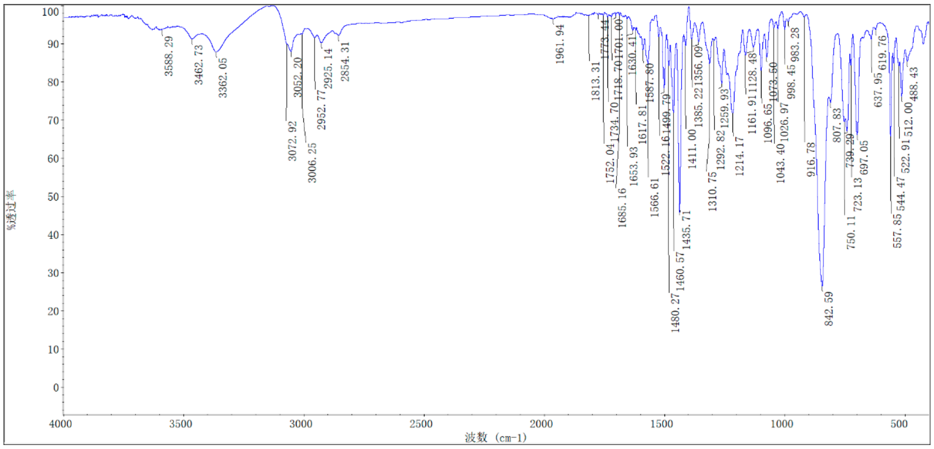

Figure S5. IR spectrum of complex **1**

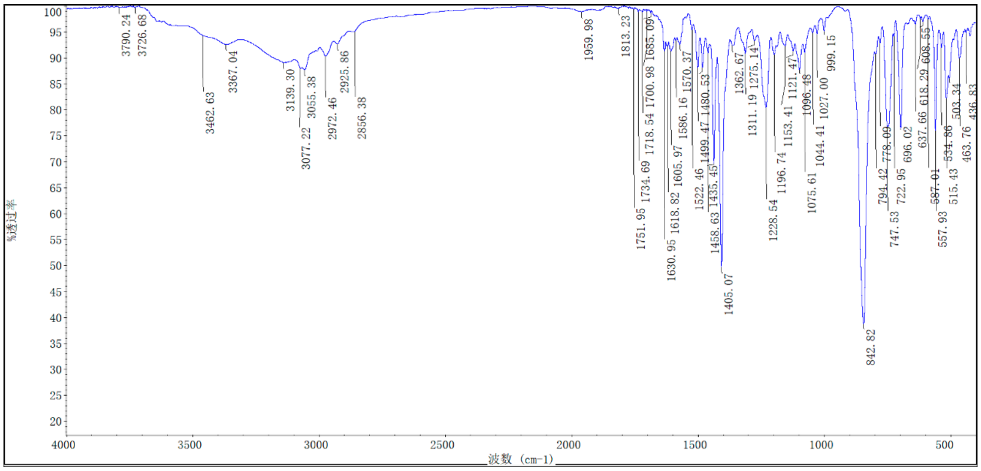

Figure S6. IR spectrum of complex **2**

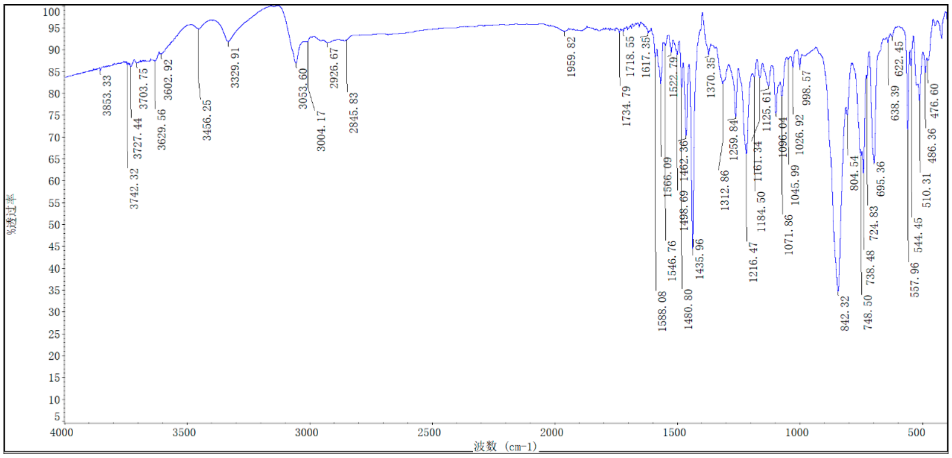

Figure S7. IR spectrum of complex **3**

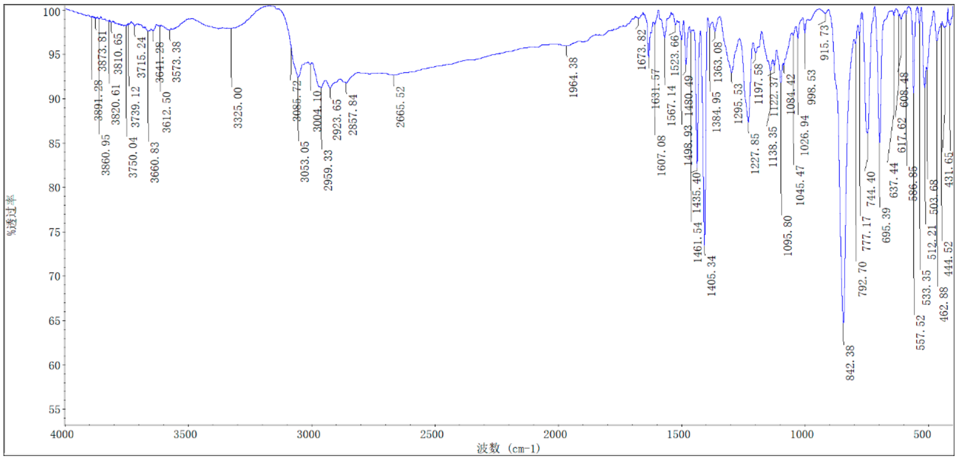

Figure S8. IR spectrum of complex **4**

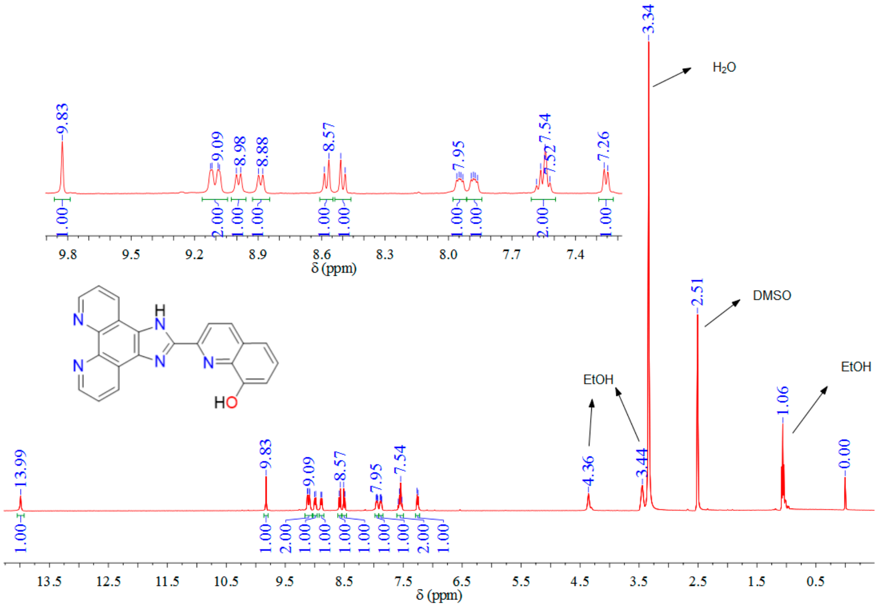

Figure S9. ^{1}H NMR of ligand ipqH_2

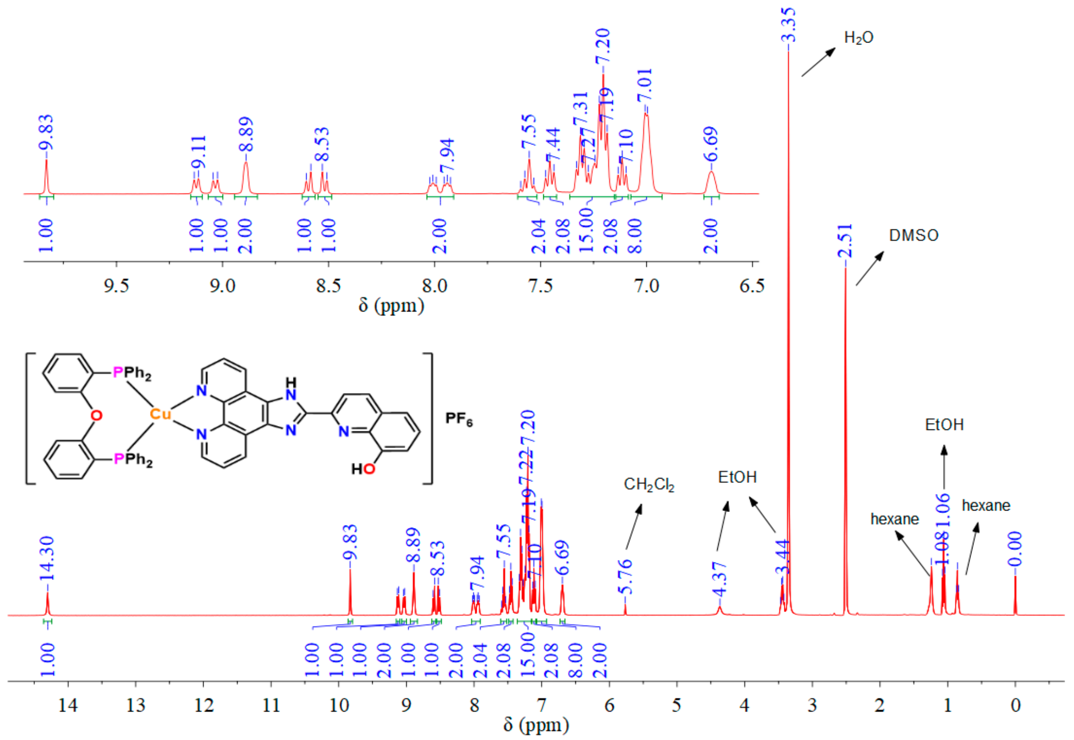

Figure S10. ^{1}H NMR of complex **1**

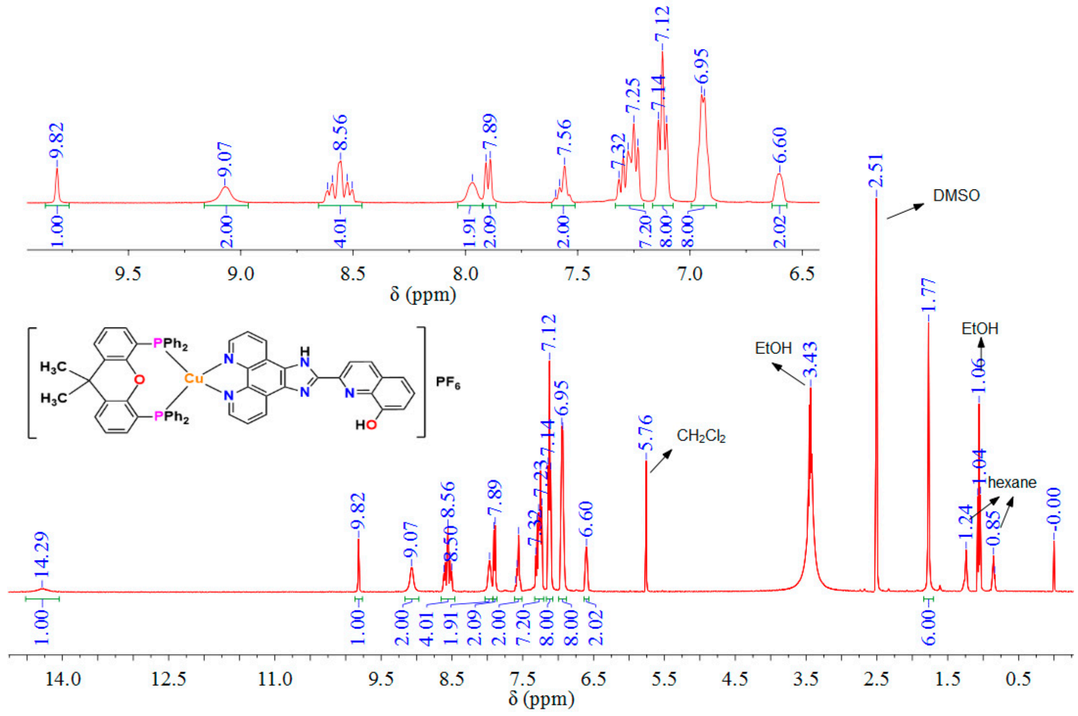

Figure S11. ^{1}H NMR of complex **2**

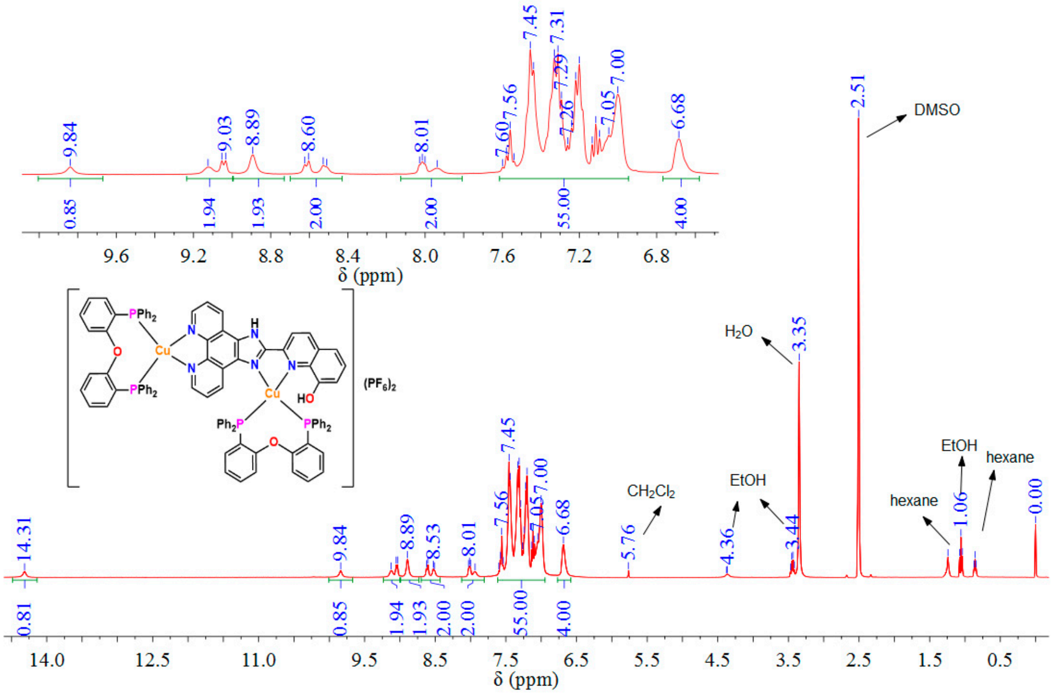

Figure S12. ^{1}H NMR of complex **3**

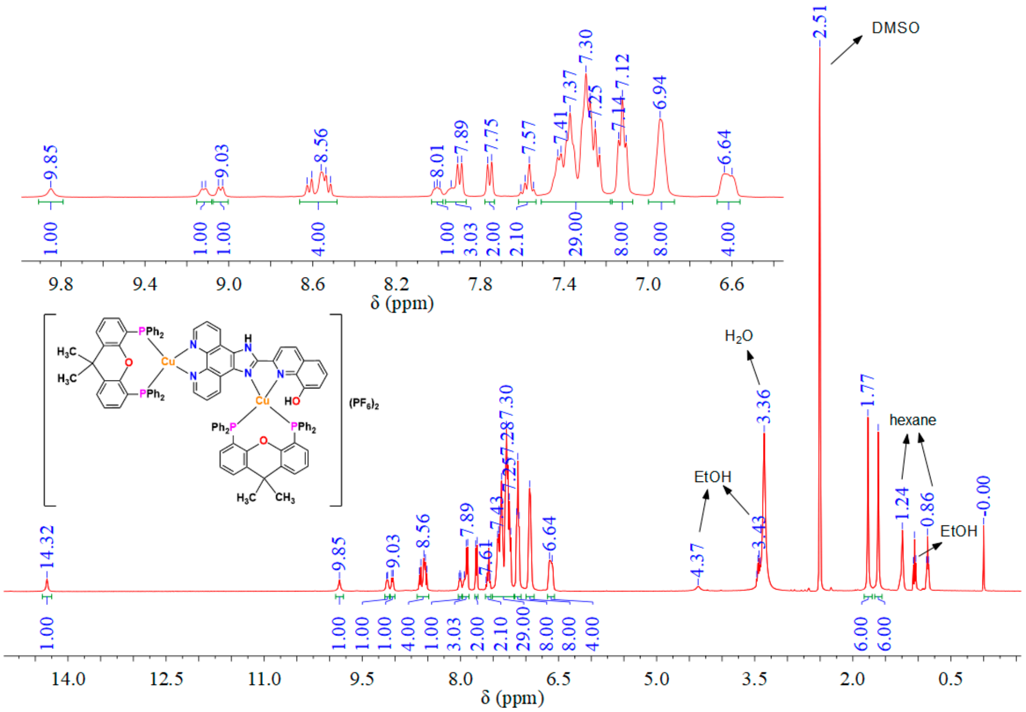

Figure S13. ^{1}H NMR of complex **4**

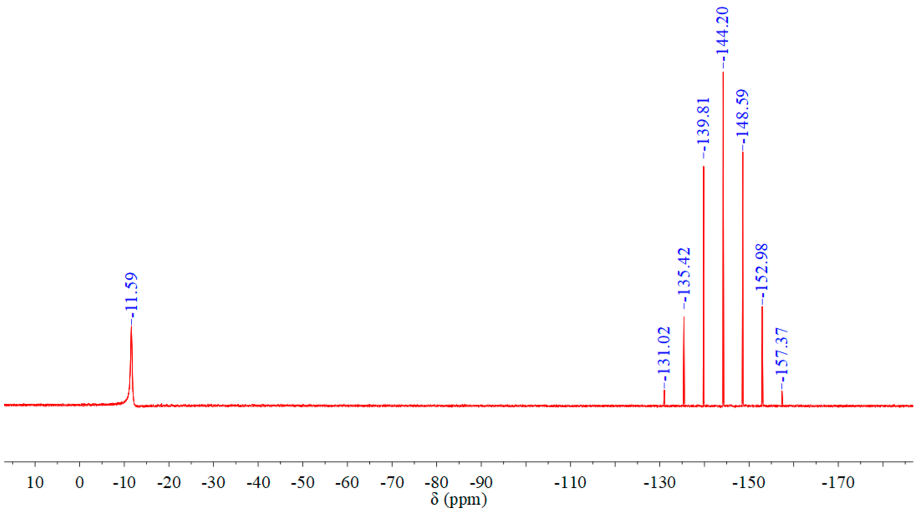

Figure S14. ^{31}P {^{1}H} NMR of complex **1**

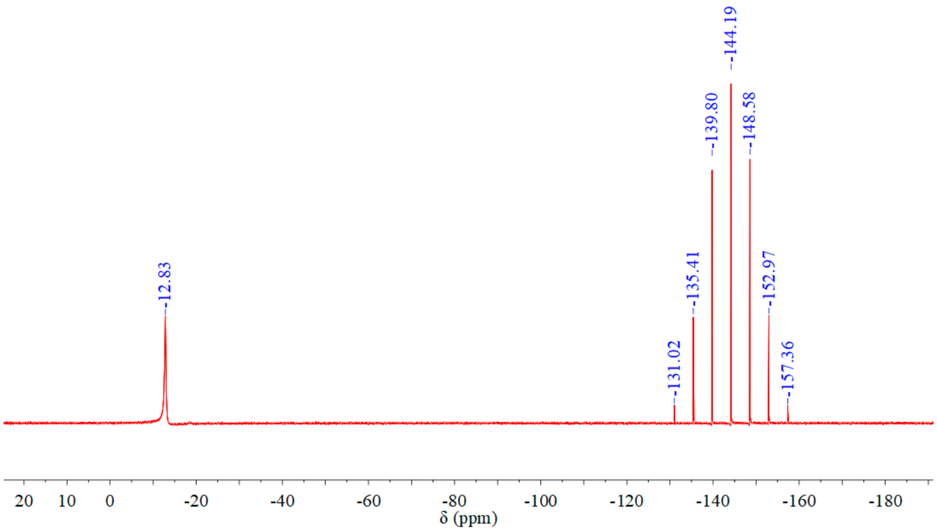

Figure S15. ^{31}P {^{1}H} NMR of complex **2**

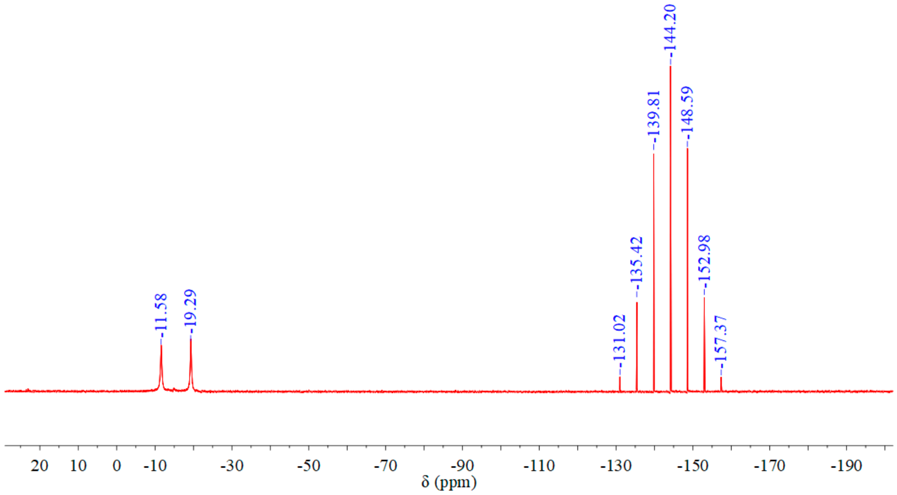

Figure S16. ^{31}P {^{1}H} NMR of complex **3**

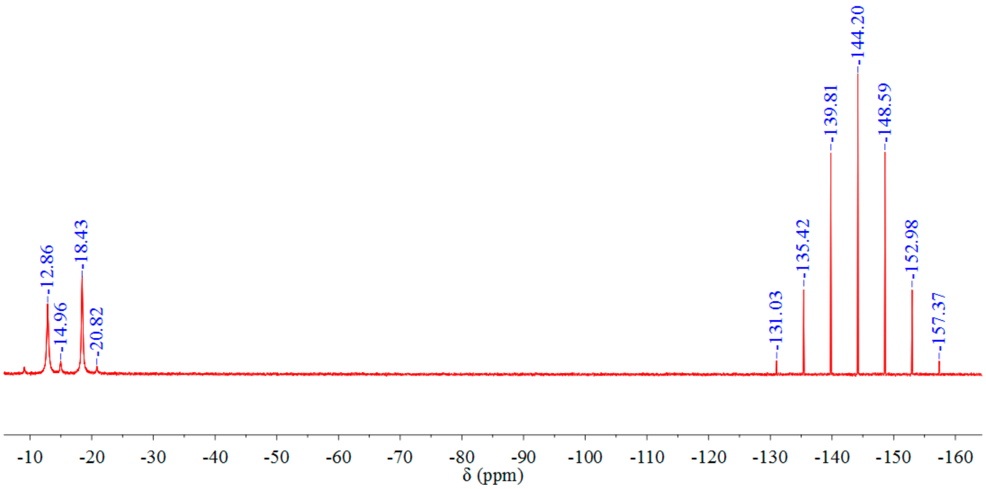

Figure S17. ^{31}P {^{1}H} NMR of complex **4**

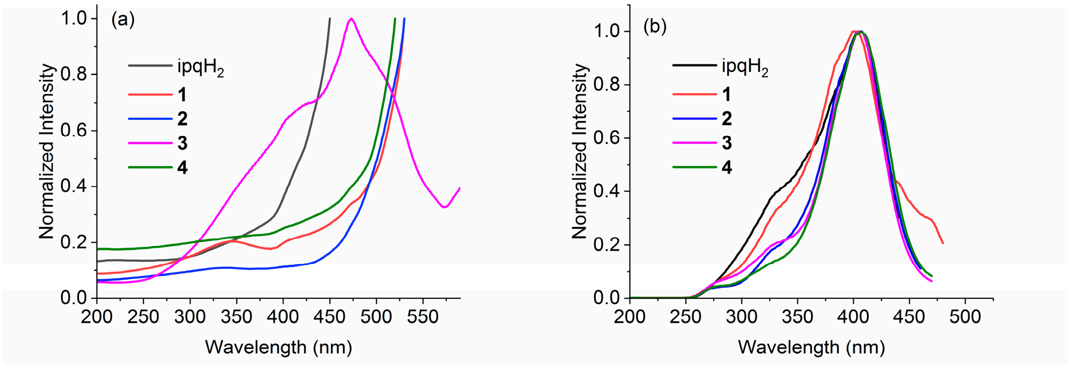

Figure S18. The excitation spectra of ipqH_2 and complexes **1–4** in the solid state (a) and in DMSO solutions (b).

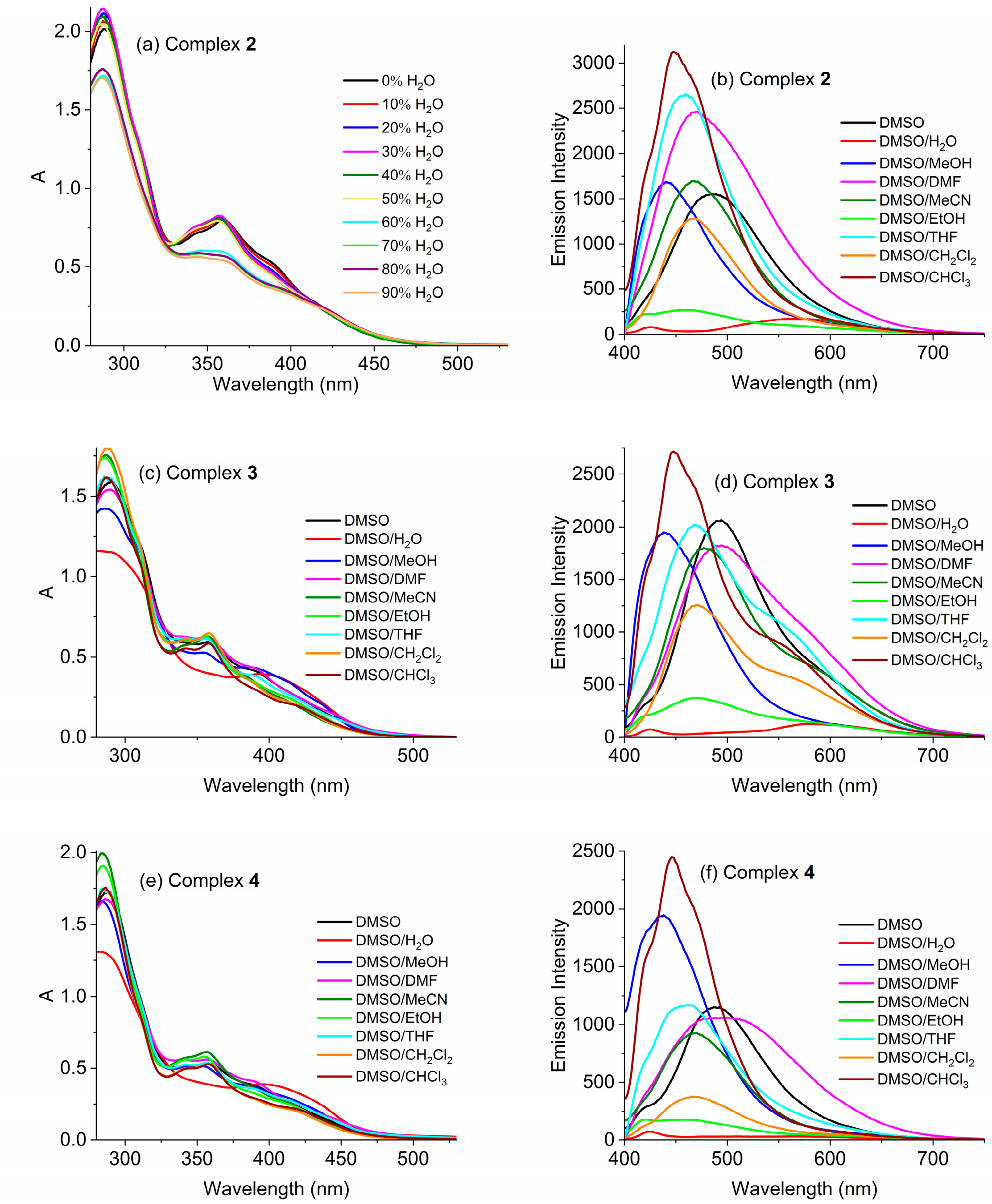

Figure S19. The UV-Vis absorption spectra and emission spectra of ligand complexes **2** (a and b), **3** (c and d) and **4** (e and f) in DMSO and different mixed solvents ($V_{DMSO}/V_{other\ solvent}$ = 1 : 9)

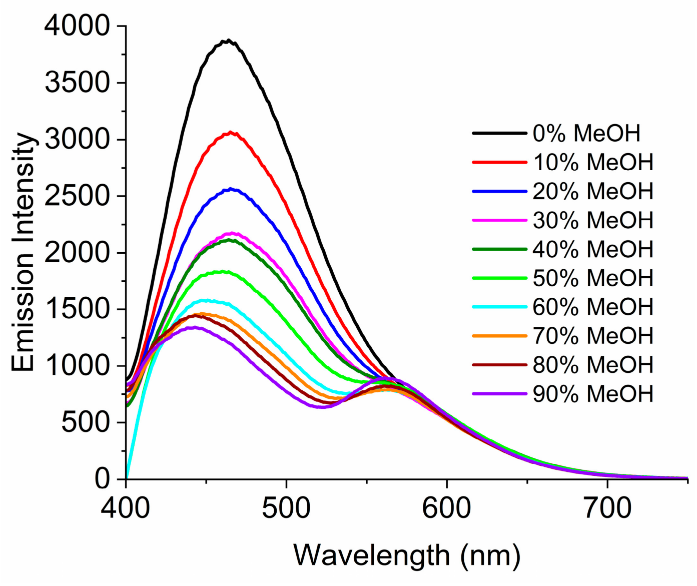

Figure S20. Emission spectra of ligand ipqH_2 in DMSO/MeOH mixed solvents with varying MeOH volumetric content.

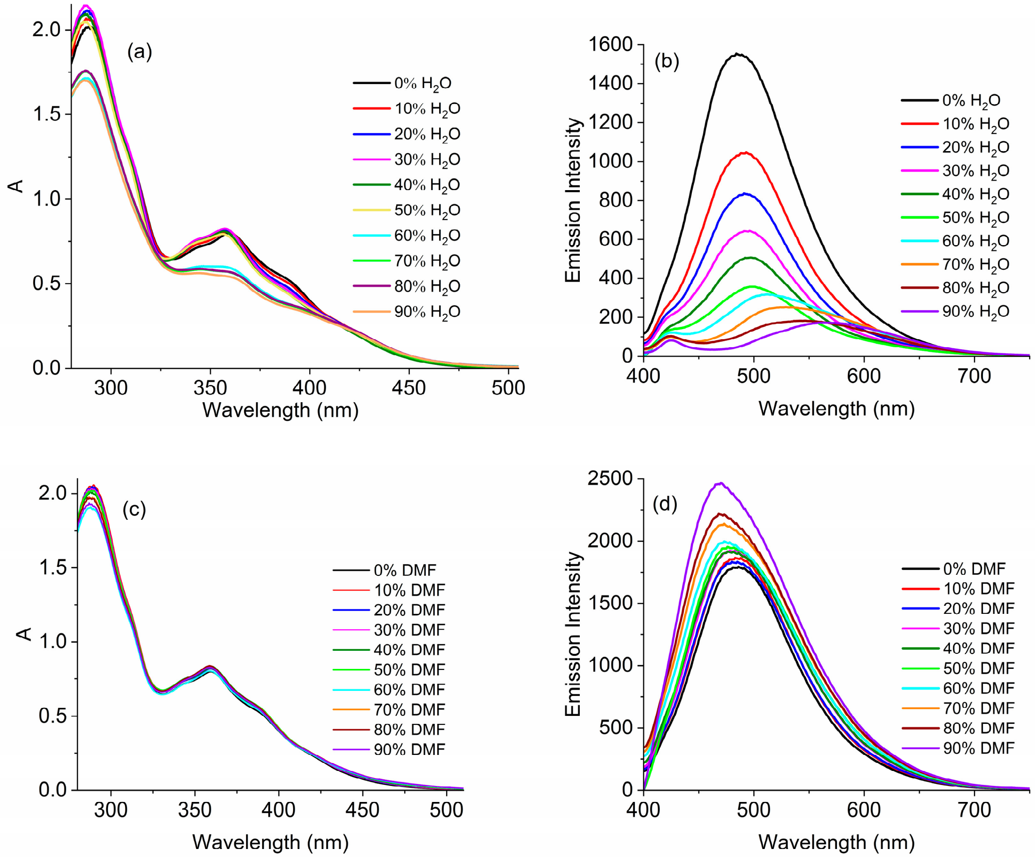

Figure S21. Variation of the UV-Vis absorption and emission spectra of complex **2** as a function of H_2O and DMF volume ratios in DMSO/ H_2O and DMSO/DMF mixed solvents.

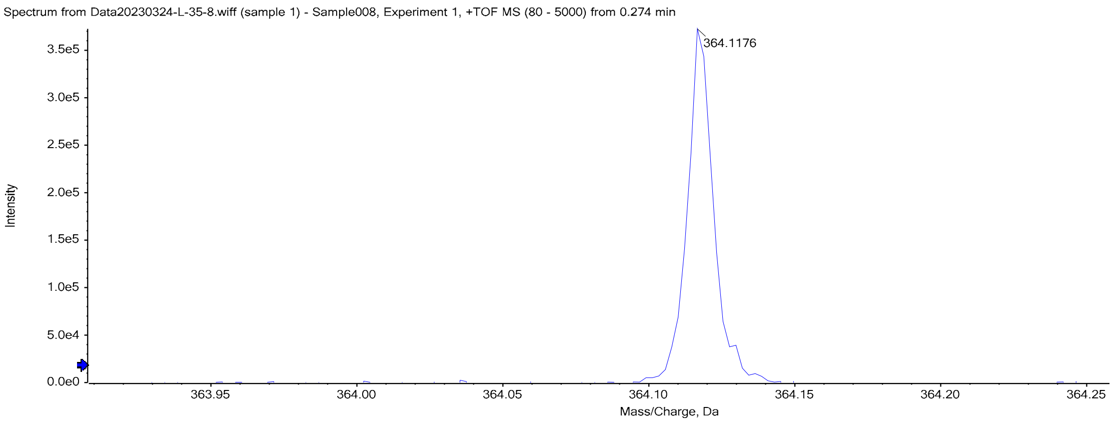

Figure S22. Experimental HRMS of [M + H^+] for ipqH_2

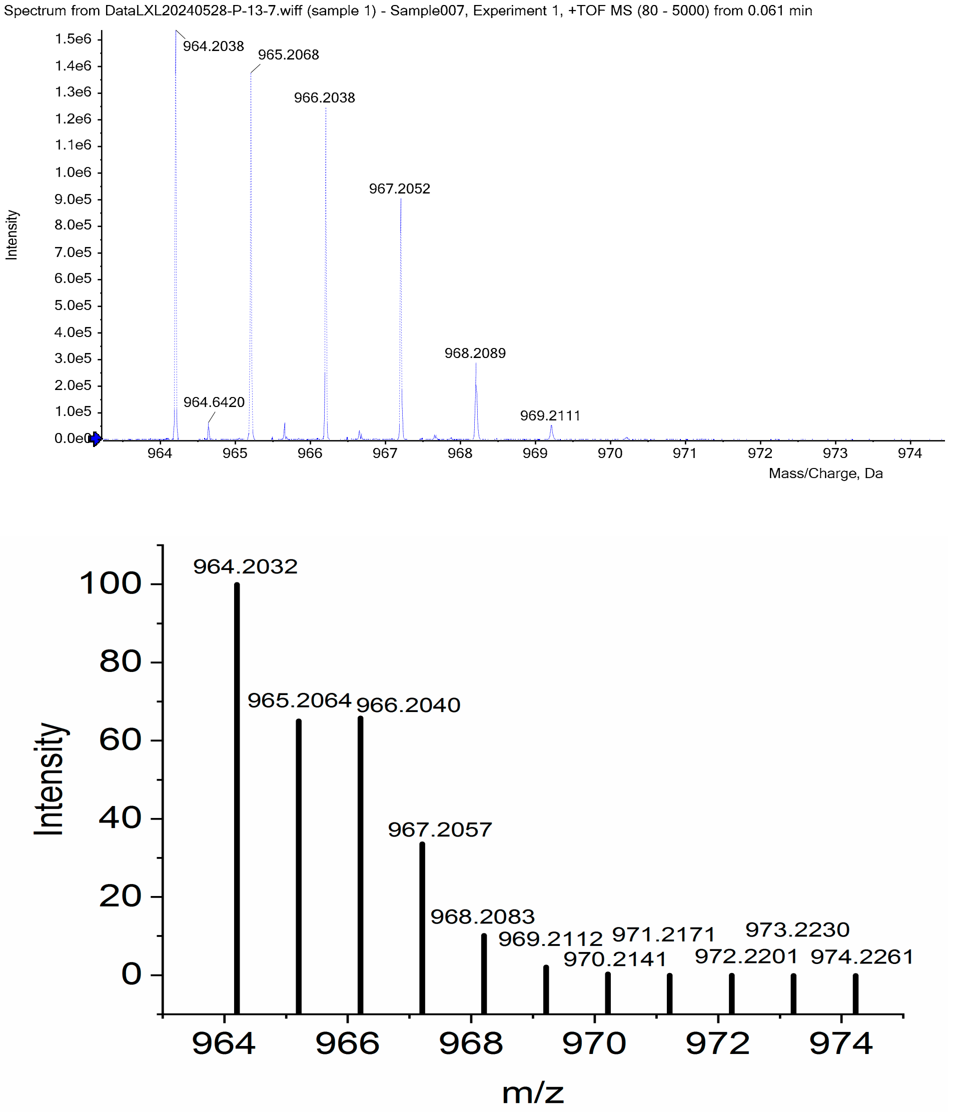

Figure S23. Experimental (blue lines) and expected (black lines) HRMS of $[Cu(ipqH_2)(POP)]^+$ for complex **1**

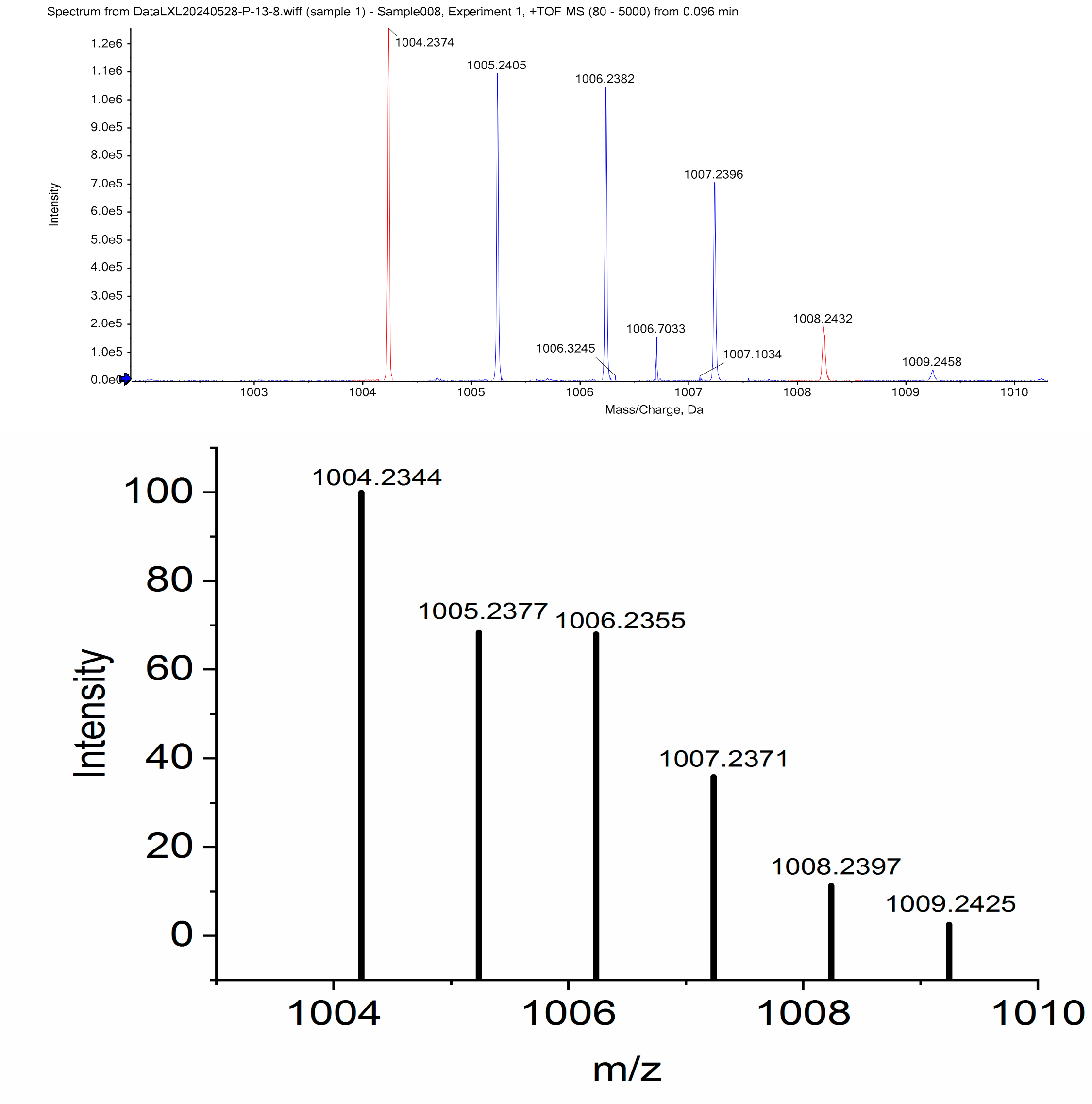

Figure S24. Experimental (red and blue lines) and expected (black lines) HRMS of [Cu(ipqH_2)(xantphos)]$^+$ for complex **2**

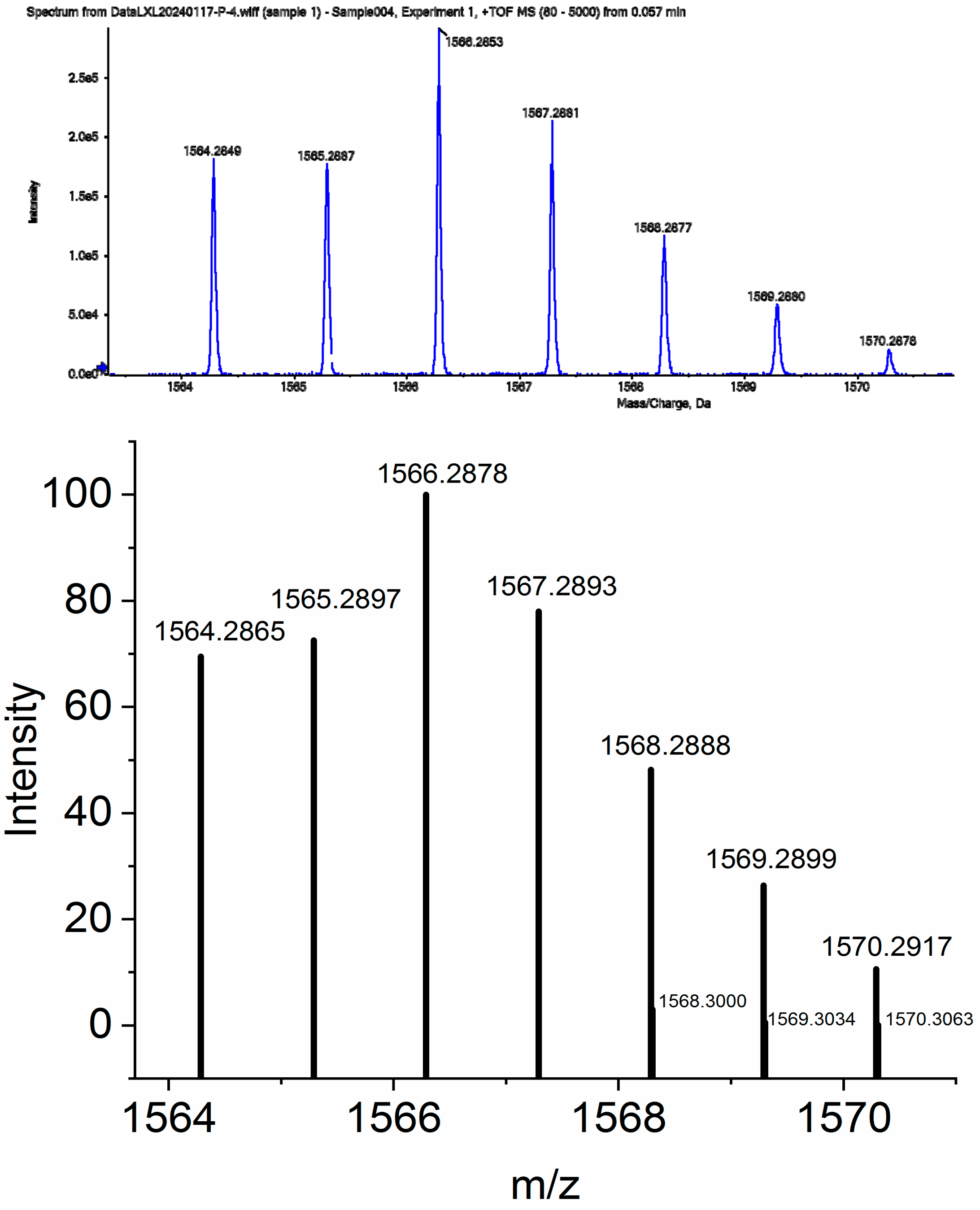

Figure S25. Experimental (blue lines) and expected (black lines) HRMS of $\{[Cu_2(ipqH_2)(POP)_2]^{2+} - H^+\}^+$ for complex **3**

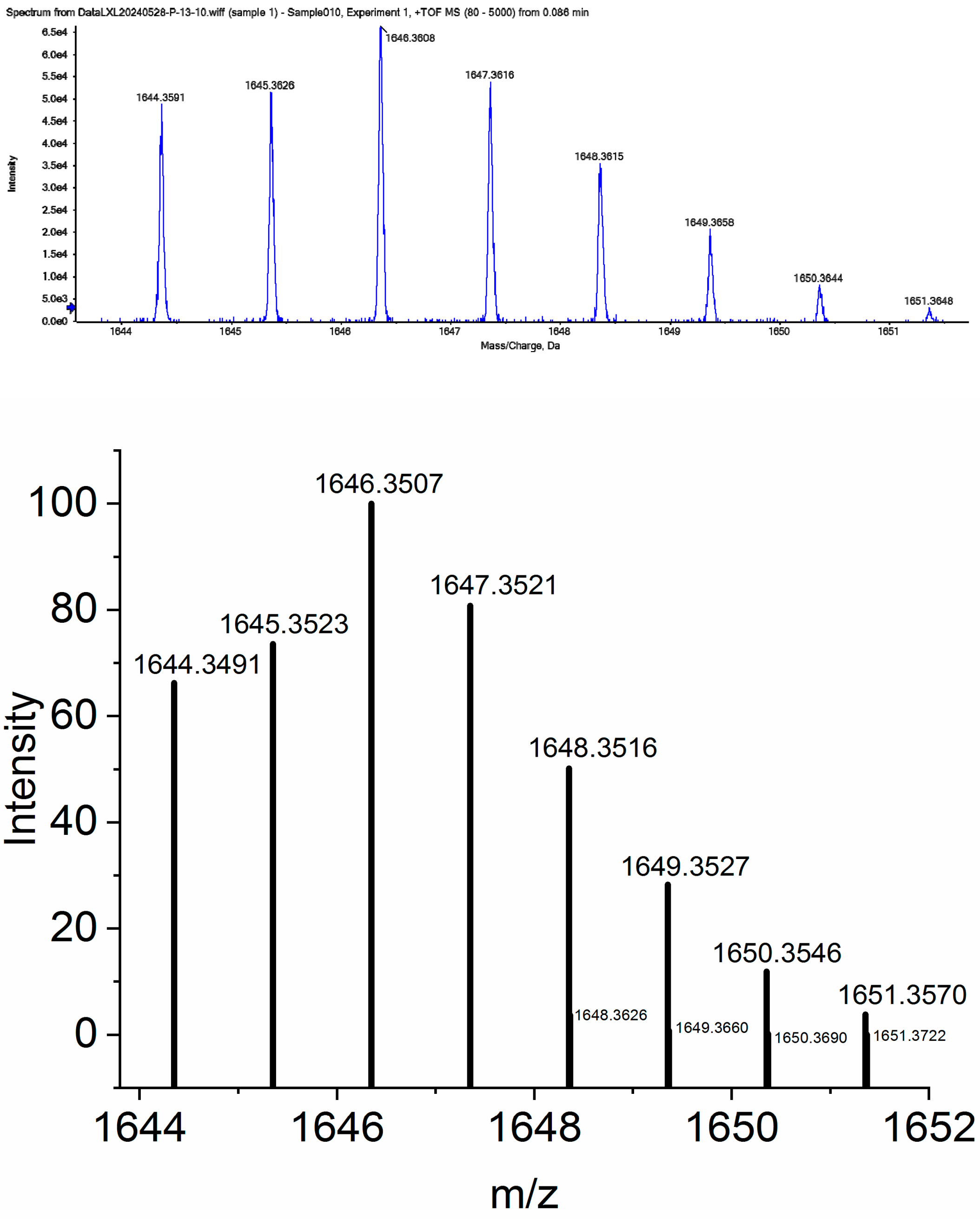

Figure S26. Experimental (blue lines) and expected (black lines) HRMS of $\{[Cu_2(ipqH_2)(xantphos)_2]^{2+} - H^+\}^+$ for complex **4**
